# N-doped nonalternant aromatic belt *via* a six-fold annulative double N-arylation†

**DOI:** 10.1039/d2sc02647c

**Published:** 2022-08-09

**Authors:** Hiroki Sato, Rie Suizu, Tomoki Kato, Akiko Yagi, Yasutomo Segawa, Kunio Awaga, Kenichiro Itami

**Affiliations:** Institute of Transformative Bio-Molecules (WPI-ITbM), Nagoya University Chikusa Nagoya 464-8602 Japan Itami@chem.nagoya-u.ac.jp; Graduate School of Science, Nagoya University Chikusa Nagoya 464-8602 Japan segawa@ims.ac.jp; Japan Science and Technology Agency (JST), PRESTO 4-1-8 Honcho Kawaguchi Saitama 332-0012 Japan; Institute for Molecular Science Myodaiji Okazaki 444-8787 Japan; Department of Structural Molecular Science, SOKENDAI (The Graduate University for Advanced Studies) Myodaiji Okazaki 444-8787 Japan; JST-ERATO, Nagoya University Itami Molecular Nanocarbon Project Chikusa Nagoya 464-8602 Japan; Integrated Research Consortium on Chemical Sciences (IRCCS), Nagoya University Japan

## Abstract

The design and synthesis of nitrogen (N)-doped molecular nanocarbons are of importance since N-doped nanocarbons have received significant attention in materials science. Herein, we report the synthesis and X-ray crystal structure of a nitrogen-inserted nonalternant aromatic belt. The palladium-catalyzed six-fold annulative double N-arylation provided an aromatic belt bearing six nitrogen atoms in one step from cyclo[6]paraphenylene-*Z*-ethenylene, the precursor of the (6,6)carbon nanobelt. The *C*_3i_-symmetric structure of the aromatic belt in the solid state was revealed using X-ray crystallography. The multistep (electro)chemical oxidation behavior of the belt, which was facilitated by the six *p*-methoxyaniline moieties, was studied, and a stable dication species was successfully identified by X-ray crystallography. The present study not only shows the unique structure and properties of the N-doped nonalternant aromatic belt but also expands the scope of accessibility of synthetically difficult belt molecules by the conventional intramolecular contraction pathway.

## Abstract 2

Nitrogen-doped nonalternant aromatic belt was synthesized *via* palladium-catalyzed six-fold annulative double N-arylation reaction. The highly symmetric structure and multistep oxidation behavior of the N-belt were confirmed.

## Graphical abstract

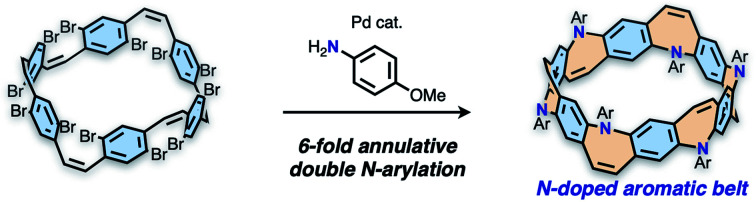

## Introduction

**Fig. 1:**
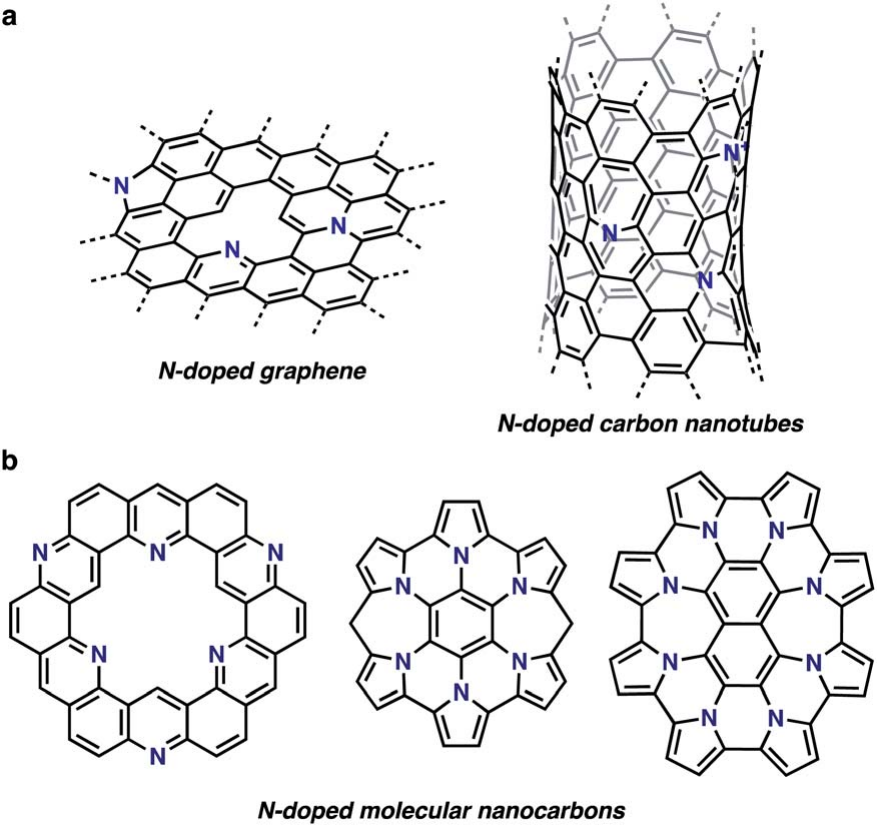
Structures of N-doped nanocarbons. (a) N-doped graphene and carbon nanotubes. (b) Examples of atomically precise N-doped molecular nanocarbons.

The research field of nitrogen (N)-doped nanocarbon materials has been expanding over the last few decades because N doping creates interactive sites in pristine nanocarbons and modifies their electronic properties (Fig. 1a).^1^ Whereas current production of intrinsically N-doped nanocarbons by post-treatment or *in situ* synthesis methods provides random dopant patterns and structures,^1*a*,*b*,*e*^ organic synthesis, in contrast, enables the formation of structurally uniform and atomically precise carbon nanostructures.^2,3^ Such nanocarbon structures, also called molecular nanocarbons, are of high importance for their use as discrete models, ligands, electronically tunable organic materials, and templates for the growth of precise large nanocarbons. For example, a number of N-doped nanographenes have been reported to date as discrete models for N-doped graphene sheets (Fig. 1b). Their unique properties and tunable electronic structures with structural uniformity have attracted considerable attention.^2*a*,3^

The tubular belt structures formed by annulated conjugated aromatic rings pose formidable synthetic challenges because of their high strain.^3^ To date, ring-shaped,^4^ planar,^5^ bowl-shaped,^6^ and triangle- or square-shaped aromatic molecules have only been reported.^7^ An N-doped aromatic belt with nonconjugated sp^3^-linkers can also release the belt strain.^8^ A recent unsuccessful synthetic endeavor of a tetraazapentacene belt also attests to the aforementioned challenge associated with the synthesis of fully conjugated N-doped aromatic belts.^9^

**Fig. 2:**
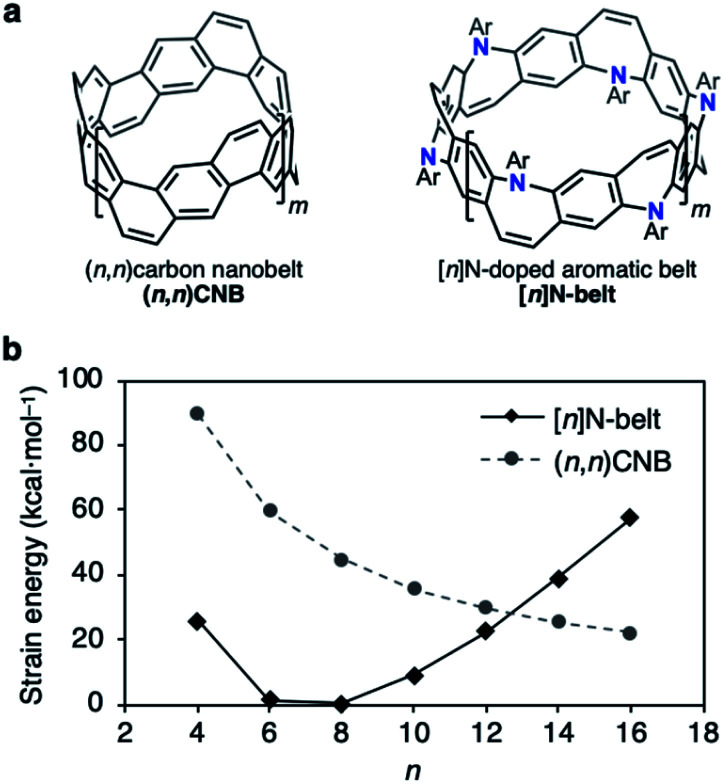
(a) Structures of (*n*,*n*)CNB and [*n*]N-belt where *n* = 2(*m* + 2). (b) Plot of the strain energies of (*n*,*n*)CNB and [*n*]N-belt (kcal mol^−1^) *versus n* calculated by the B3LYP/6-31G(d) level of theory; Ar = Ph (see the ESI for details†).

Herein, we report the synthesis of an N-doped aromatic belt *via* annulative amination reactions of the *Z*-stilbene-based macrocyclic intermediate 1 (Fig. 2). The crystal structure and optical properties of 2 are presented. We also report structural determination of the corresponding dication salts of 2 obtained *via* chemical oxidation.

## Results and discussion

The difficulty of accessing belt molecules lies in the final ring-closing step in which a particular precursor forms the highly strained belt structures. Thus, available synthetic strategies are rather limited. In our previous work, fully fused carbon nanobelts (CNBs, Fig. 2a, left)^10*a*,*b*^ were synthesized *via* intramolecular reductive or oxidative C–C bond-forming reactions in the final annulation step. On the other hand, the strategy of inserting belt components intermolecularly during annulation events (N-belt, Fig. 2a, right) has not yet been studied, although this approach has potential benefits regarding substrate flexibility and variety. Based on the recent progress in Buchwald–Hartwig amination reactions, we speculated an intermolecular synthetic strategy for N-doped belt synthesis *via* annulative C–N bond formation reactions of zigzag macrocycles.

To estimate its synthetic difficulty, the strain energy of the N-belt was calculated. The calculation of the strain energy of [*n*]N-belt was performed by using density functional theory (DFT) B3LYP/6-31G(d) level of theory with hypothetical homodesmotic reactions (see Fig. S4 in the ESI for details†). Fig. 2b shows the strain energy of [*n*]N-belt and (*n*,*n*)CNB. It was clearly seen that the size-dependency of the strain energy of [*n*]N-belt was different from that of (*n*,*n*)CNB.^10*a*^ Because of the nonplanar structure of 7-membered rings in [*n*]N-belt, [6]- and [8]N-belt have almost no strain (1.3 and 0.4 kcal mol^−1^, respectively), whereas small (*n* = 4) and large [*n*]N-belts (*n* = 10–16) have high strain energy. According to these analyses, [6]- and [8]N-belt would be suitable targets for the synthesis of N-doped belt molecules.

**Scheme 1:**
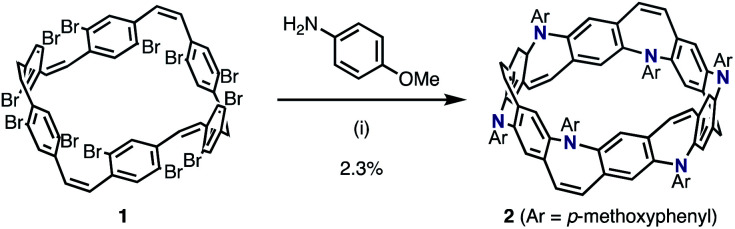
Synthesis of N-doped aromatic belt 2. Reaction conditions: (i) 1 (1.0 equiv.) *p*-methoxyaniline (6.0 equiv.), Pd(dba)_2_ (0.60 equiv.), PMe(^*t*^Bu_2_)·HBF_4_ (1.2 equiv.), NaO^*t*^Bu (30 equiv.), *m*-xylene, 140 °C, 8 h.

We envisioned that the *Z*-stilbene-based macrocycle 1 could afford the target [6]N-belt in one step by the 12-fold annulative Buchwald–Hartwig reactions^11^ (or the so-called annulative double *N*-arylation reactions^12^). *Z*-stilbene-based macrocyclic precursor 1 was prepared following our previous report.^10*a*^ Next, palladium-catalyzed amination reactions between 1 and aniline were performed. In the first attempt, debrominated, hydroaminated, and over-/less-aminated ring products were detected by mass spectrometry but not the desired belt molecules. Several amines such as 4-(dimethylamino)aniline, ethyl 4-aminobenzoate, 4-aminopyridine, and benzylamine were screened, aiming at the reduction of these undesired products. Through this screening, it was found that *p*-methoxyaniline afforded the desired belt molecule 2 (the methoxy derivative of [6]N-belt), probably because the electron-donating methoxy groups promoted the reductive elimination. Among the various conditions tested, Pd(dba)_2_ (dba = dibenzylideneacetone) and PMe(^*t*^Bu)_2_·HBF_4_ salt as the catalyst and ligand, respectively, furnished the desired compound most efficiently. In particular, the reaction between 1 and *p*-methoxyaniline using 0.60 equiv. Pd(dba)_2_, 1.2 equiv. PMe(^*t*^Bu)_2_·HBF_4_, and 30 equiv. of NaO^*t*^Bu in *m*-xylene at 140 °C for 8 h provided 2 in 2.3% isolated yield (Scheme 1). The structure of nanobelt 2 was fully characterized by ^1^H and ^13^C NMR spectroscopies, MALDI-TOF MS, and X-ray diffraction.

**Fig. 3:**
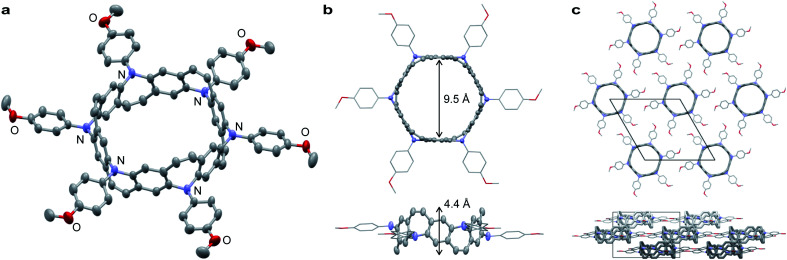
X-ray crystal structure of N-doped aromatic belt 2. (a) Structure of 2 with 50% thermal ellipsoids; co-crystallized *n*-pentane molecules are omitted for clarity. One-sixth of the entire structure constitutes a crystallographically independent unit. (b) Size and height of 2. (c) Packing structure of 2: top view (top) and side view (bottom).

Belt 2 is a yellow powder that can be dissolved in halogenated solvents. A single crystal of 2 was obtained by the slow diffusion of *n*-pentane into a 1,2-dichloroethane solution. The crystal structure of 2 belongs to the trigonal space group *R*3. As shown in Fig. 3a and b, 2 exhibits a tubular belt structure with approximately 9.5 Å pore and 4.4 Å height. Six *p*-methoxyphenyl substituents project radially out from the center of the belt. The sum of the three C–N–C angles around N (*Σ*_N_) is 351.6° indicating that the nitrogen atoms are almost sp^2^-hybridized. A disordered *n*-pentane molecule was incorporated in the cavity of the belt. The packing structure of 2 revealed a columnar structure with side-by-side packing, and there were no notable π–π interactions between neighboring molecules (Fig. 3c).

**Fig. 4:**
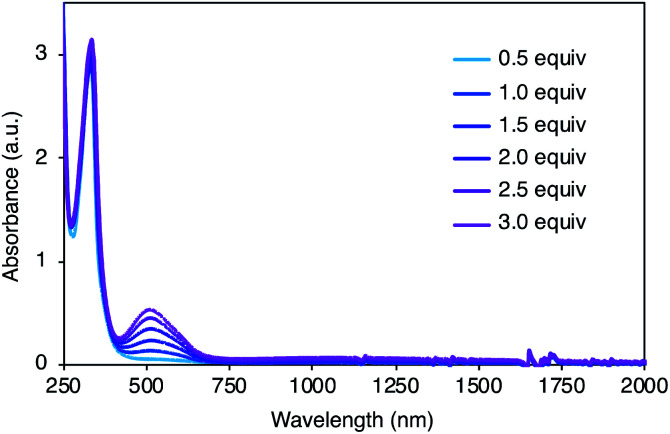
UV-Vis-NIR absorption spectra of 2 (5.0 × 10^−5^ M) with AgSbF_6_ (0.5–3.0 equiv.) in dichloromethane.

To gain insight into the redox properties of 2, its chemical and electrochemical oxidation experiments were performed, and ultraviolet-visible-near infrared (UV-Vis-NIR) absorption measurements were conducted. Neutral 2 exhibited a single absorption peak at 334 nm and no obvious fluorescence upon irradiation of 334 nm light. With the addition of AgSbF_6_ as an oxidant, a new absorption peak appeared at around 520 nm (Fig. 4), and the peak intensity continuously increased for additions of up to 3.0 equiv. of AgSbF_6_. With more than 3.0 equiv. of the oxidant, insoluble solids started to precipitate, resulting in weaker absorption bands (Fig. S2†), probably due to intermolecular oxidative coupling reactions. No other peak, except the one at 520 nm, was observed during the incremental addition of the oxidant. Cyclic voltammetry (CV) and differential pulse voltammetry (DPV) measurements were carried out in dichloromethane (0.1 M ^*n*^Bu_4_NPF_6_) (Fig. S3†). CV analysis showed a reversible redox process, and DPV revealed multiple oxidation steps including *E*_Ox_ = 0.42 and 0.54 [*E*_Ox_*vs.* Fc/Fc^+^].

**Fig. 5:**
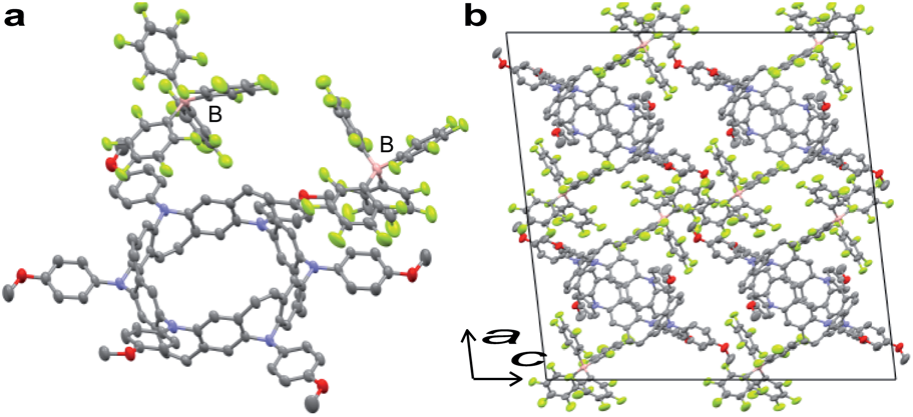
X-ray crystal structure of 2^2+^·2[B(C_6_F_5_)_4_]^−^. (a) Structure of 2^2+^·2[B(C_6_F_5_)_4_]^−^ with 50% thermal ellipsoids; co-crystallized solvent molecules (*i.e.*, *n*-hexane and dichloromethane) are omitted for clarity. Half of the entire structure constitutes a crystallographically independent unit. (b) The packing structure of 2^2+^·2[B(C_6_F_5_)_4_]^−^.

Stoichiometrically controlled chemical oxidation of 2 and structural characterization of oxidized species were attempted. Oxidants with different counter anions and varying oxidant stoichiometry were screened. It was found that the stabilities of the cations were highly dependent on the counter anions. The purple color of the solutions derived from the radical cations disappeared within a short period for BF_4_^−^ or PF_6_^−^ counter anions, whereas the solutions remained purple for a while in the case of SbF6^−^ or SbCl6^−^. Treatment of 2 with 2 equiv. of AgB(C_6_F_5_)_4_ in dichloromethane and slow diffusion in *n*-hexane at −30 °C over a period of 1 week resulted in the formation of thick purple single crystals of the N-doped nanobelt dication salt 2^2+^·2[B(C_6_F_5_)_4_]^−^. The structure of 2^2+^·2[B(C_6_F_5_)_4_]^−^ was determined by X-ray diffraction (Fig. 5). The space group was monoclinic *I*2/*a*. Unlike the neutral compound, where one-sixth of the molecule is an asymmetric unit, half of the molecule is crystallographically independent of the dication compound, and the belt structure contracts uniaxially. Because of the instability of 2^2+^ in solution, spectral analysis could not be performed.

**Fig. 6:**
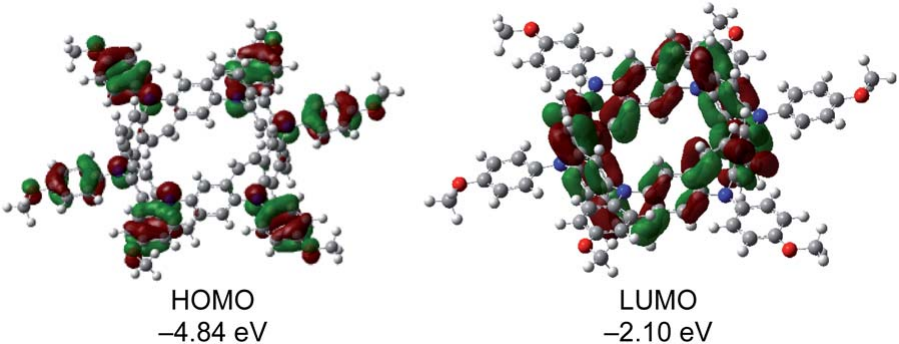
Frontier molecular orbitals (isovalue = 0.02) of 2 and their calculated energies.

To further investigate the electronic nature of 2, DFT calculations were carried out at the B3LYP/6-31 G (d) level. The calculated optimal structure of 2 (Fig. S5†) matches well with the experimental results obtained by X-ray analysis. Notably, DFT studies revealed that the energies of all orbitals between HOMO and HOMO−5 lie within a narrow range of 0.10 eV (Fig. S6†). Furthermore, the electron density on the HOMO was localized on the *p*-methoxyphenylamine moieties, while that on the LUMO was localized on the central belt (Fig. 6). DFT calculations performed on the oxidized species revealed that the optimal structures of 2^*n*+^ in different oxidation states (*n* = 1–3) were similar (Fig. S7†) and spins were localized on the *p*-methoxyphenylamine moieties (Fig. S8†). Time-dependent DFT calculations of 2 and the cations also supported the experimental results (Table S2†). The absorption peak of 2 at 334 nm can be assigned to the HOMO−6 → LUMO+1 (360.6 nm, *f* = 0.9802) and HOMO−6 → LUMO+2 (360.6 nm, *f* = 0.9802) transitions at the belt center. The calculations for 2^n+^ show similar absorption bands in different oxidation states around 550 nm (Fig. S9†), which may correspond to the observed absorption band at 520 nm in the chemical oxidation experiment.

## Conclusions

We successfully synthesized a novel class of N-doped aromatic belts *via* a six-fold annulative double *N*-arylation reaction. This new synthetic strategy can expand the scope of accessibility of synthetically difficult belt molecules using the conventional intramolecular contraction pathway. N-doped belt 2 was chemically oxidized, and the generated diradical species 2^2+^·2[B(C_6_F_5_)_4_]^−^ was successfully characterized. Further studies on the detailed electronic and magnetic properties of 2^*n*+^ are required for their future applications.

## Author contributions

K. I., Y. S., and H. S. conceived the concept and prepared the manuscript with feedback from the other authors. H.S. performed the synthetic experiments. Y. S. and R. S. performed the X-ray crystallographic measurements. H. S., R. S., T. K., and A. Y. performed the photophysical and electrochemical measurements. A. Y. and K. A. provided critical comments.

## Conflicts of interest

There are no conflicts to declare.

## Supplementary Material

SC-013-D2SC02647C-s001

SC-013-D2SC02647C-s002
