# Supplementary material for: N-doped nonalternant aromatic belt via a six-fold annulative double N-arylation
Source: Chem Sci. 2022 Aug 9;13(34):9947–51. doi: 10.1039/d2sc02647c (PMC9430306; doi:10.1039/d2sc02647c)
Supplement: SC-013-D2SC02647C-s001 [file SC-013-D2SC02647C-s001.pdf]

Electronic Supplementary Material (ESI) for Chemical Science.
This journal is © The Royal Society of Chemistry 2022

Supporting Information

N-doped Nonalternant Aromatic Belt via a Six-fold Double N-Arylation

Hiroki Sato,[1] Rie Suizu,[2,3] Tomoki Kato,[2] Akiko Yagi,[1] Yasutomo Segawa,[2,4,5,6,*] Kunio Awaga,[2,7] and Kenichiro Itami[1,2,6,7,*]

[1] Institute of Transformative Bio-Molecules (WPI-ITbM), Nagoya University, Chikusa, Nagoya 464-8602, Japan
[2] Graduate School of Science, Nagoya University, Chikusa, Nagoya 464-8602, Japan
[3] Japan Science and Technology Agency (JST), PRESTO, 4-1-8 Honcho, Kawaguchi, Saitama, 332-0012, Japan
[4] Institute for Molecular Science, Myodaiji, Okazaki, 444-8787, Japan.
[5] Department of Structural Molecular Science, SOKENDAI (The Graduate University for Advanced Studies), Myodaiji, Okazaki, 444-8787, Japan.
[6] JST-ERATO, Itami Molecular Nanocarbon Project, Nagoya University, Chikusa, Nagoya 464-8602, Japan
[7] Integrated Research Consortium on Chemical Sciences (IRCCS), Nagoya University, Chikusa, Nagoya 464-8602, Japan
*E-mail: segawa@ims.ac.jp (Y.S.), itami@chem.nagoya-u.ac.jp (K.I.)

Table of Contents

1. Instrumentation and chemicals S2–3
2. Synthesis of N-belt **2** and $\mathbf{2}^{2+}\cdot 2[B(C_6F_5)_4]^-$ S4–7
3. X-ray crystallography of **2** and $\mathbf{2}^{2+}\cdot 2[B(C_6F_5)_4]^-$ S8
4. Photophysical measurements of N-belt **2** S9
5. Electrochemical measurements of N-belt **2** S11
6. Computational study S12–32
7. References S33

1. Instrumentation and chemicals

Materials. Unless otherwise noted, all reactions were performed with dry solvents under an atmosphere of nitrogen in dried glassware with standard vacuum-line techniques. Materials were obtained from commercial suppliers and used without further purification. Silver salt $Ag[B(C_6F_5)_4]$ was prepared following the known procedure.[1] Macrocycle precursor **1** was prepared following our previous paper.[2]

Dichloromethane (DCM) for reactions was purified by passing through a solvent purification system (Glass Contour). All work-up and purification procedures were carried out with reagent-grade solvents in air. Analytical thin-layer chromatography (TLC) was performed using E. Merck silica gel 60 F254 coated plates (0.25 mm). The developed chromatogram was analyzed by UV lamp (254 nm and 365 nm). Flash column chromatography was performed with E. Merck silica gel 60 (230–400 mesh). Membrane filtration was performed with Merck Milipore Ltd. Millex® LH 0.45 μm. Preparative HPLC was performed using YMC Multiple preparative HPLC LC-Forte/R instrument equipped with YMC-Actus SIL 250 × 30.0 mm S-5 μm 12nm column.

Measurements. Melting points were measured on a MPA100 Optimelt automated melting point system. High-resolution mass spectra (HRMS) were determined on a JEOL JMS-S3000 SpiralTOF (MALDI-TOF MS) using polyethyleneglycol mixture (PEG) as internal standards and *trans*-2-[3-(4-*tert*-butylphenyl)-2-methyl-2-propenylidene] malononitrile (DCTB) as matrix with NaI as cationizing agent. Nuclear magnetic resonance (NMR) spectra were recorded on JEOL spectrometers (JNM-ECA-600: ^{1}H 600 MHz, ^{13}C 150 MHz;, JEOL ECA 500II: ^{1}H 500 MHz, ^{13}C 125 MHz). Chemical shifts for ^{1}H NMR are expressed in parts per million (ppm) relative to $CHDCl_2$ (δ 5.32 ppm). ^{13}C NMR spectra were run using a proton-decoupled pulse sequence. Chemical shifts for ^{13}C NMR are expressed in ppm relative to CD_2Cl_2 (δ 54.0 ppm). The following abbreviations were used to explain the multiplicities: s (singlet), d (doublet), t (triplet), sept (septuplet), m (multiplet). Coupling constants, *J*, are reported in Hz and with an accuracy of one unit of the last digit.

For photophysical measurements, dilute solutions in degassed spectral grade dichloromethane in a 1 cm square quartz cell were used. UV–vis absorption spectra were recorded on a Shimadzu UV-3510 spectrometer with a resolution of 0.2. UV-Vis-NIR absorption spectra were recorded on a JASCO UV V-570 spectrometer with a resolution of 2 nm between 250 nm to 2500 nm. Fluorescence spectra were recorded with a Shimadzu RF6000 spectrofluorometer using a 0.1

nm bandwidth in both excitation and emission. Absolute FL quantum yields were determined with the same instrument equipped with a calibrated integrating sphere. Electron paramagnetic resonance (EPR) spectra were recorded on JEOL ESR JES TE-200 instruments using quartz Schlenk tube filled with Ar.

2. Synthesis of N-belt (2) and dications salt $2^{2+}\cdot 2[B(C_6F_5)_4]^-$

Synthesis of N-belt **2** *via* a palladium catalyzed 6-fold double *N*-arylation

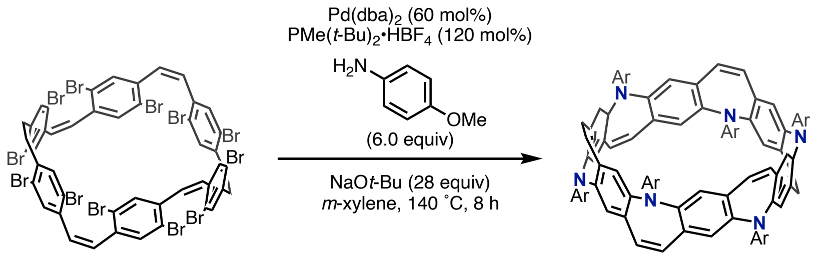

1 **2** (Ar = *p*-methoxyphenyl)

To a dry Schlenk tube equipped with argon containing a magnetic stirring bar were added **1** (122.0 mg, 78.1 µmol), *p*-anisidine (56.7 mg, 460 µmol), $Pd(dba)_2$ (26.4 mg, 45.9 µmol), $PMe(^tBu)_2\cdot HBF_4$ (23.0 mg, 92.7 µmol), and NaO^tBu (211 mg, 2.19 mmol). Then, the flask was replaced by argon gas with a balloon. To this flask was added *m*-xylene (4.8 mL), and Schlenk tube was capped. After stirring at 140 °C for 8 h, 3.0 g silica gel was added and the solvent was removed by rotary evaporation. After removal of the solvent, the product on silica was put on silica pad and eluted with toluene/EtOAc = 90:10. Then, the filtrate was concentrated and further purified by preparative HPLC (42 mL/min, toluene/EtOAc = 95:5) to afford N-belt **2** (2.4 mg, 1.8 µmol, 2.3%) as a yellow powder. ^{1}H NMR (600 MHz, CD_2Cl_2) δ 7.36 (s, 12H), 6.77 (s, 12H), 6.69 (d, J = 8.9 Hz, 12 H), 6.44 (d, J = 8.9 Hz, 12H), 3.71 (s, 18H). ^{13}C NMR (150 MHz, CD_2Cl_2) δ 153.3, 143.02, 142.97, 138.8, 132.2, 130.7, 114.6, 113.6, 56.1. HRMS (MALDI-TOF, DCTB matrix): Exact mass calculated for $C_{90}H_{66}N_6O_6$ ($[M]^+$): m/z = 1326.5038, mass found: 1326.5020; melting point: decomposed ~210 ºC. R_f = 0.2–0.4 (dichloromethane).

^{1}H NMR spectrum of **2**

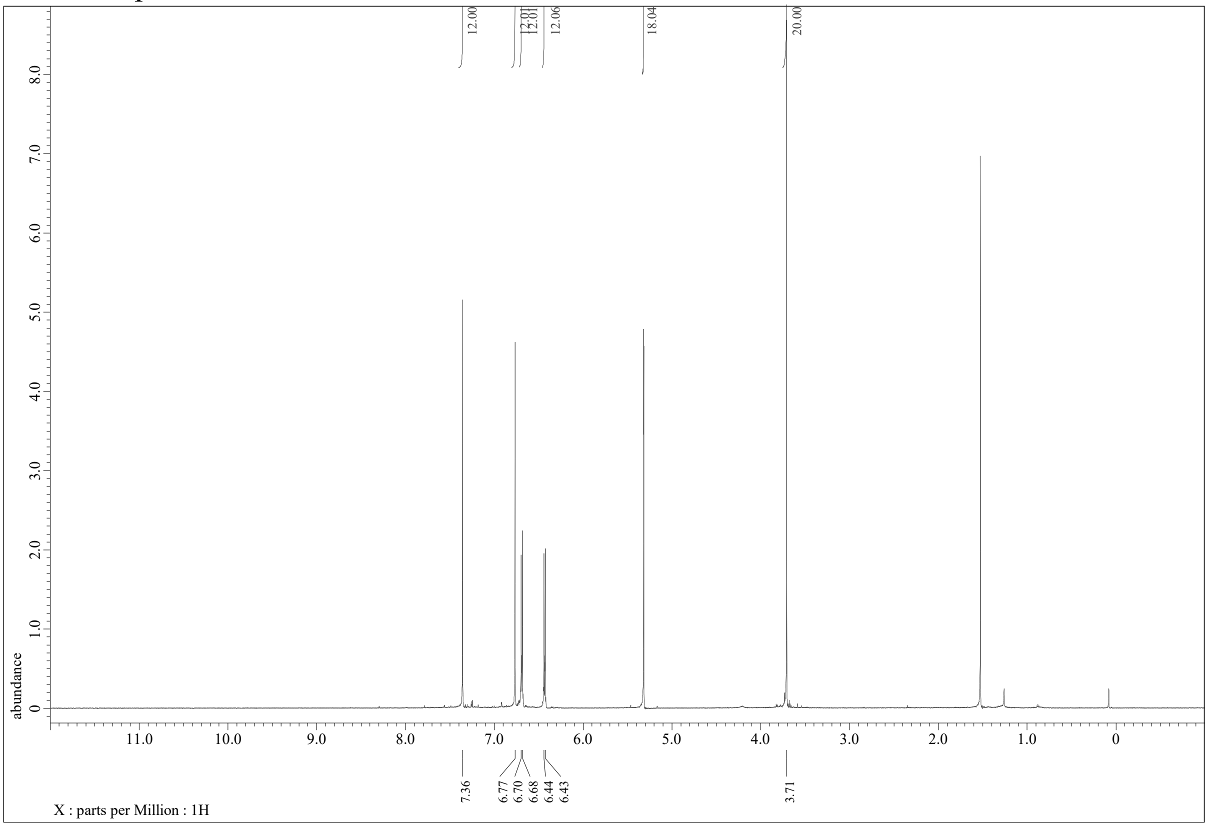

^{13}C NMR spectrum of **2**

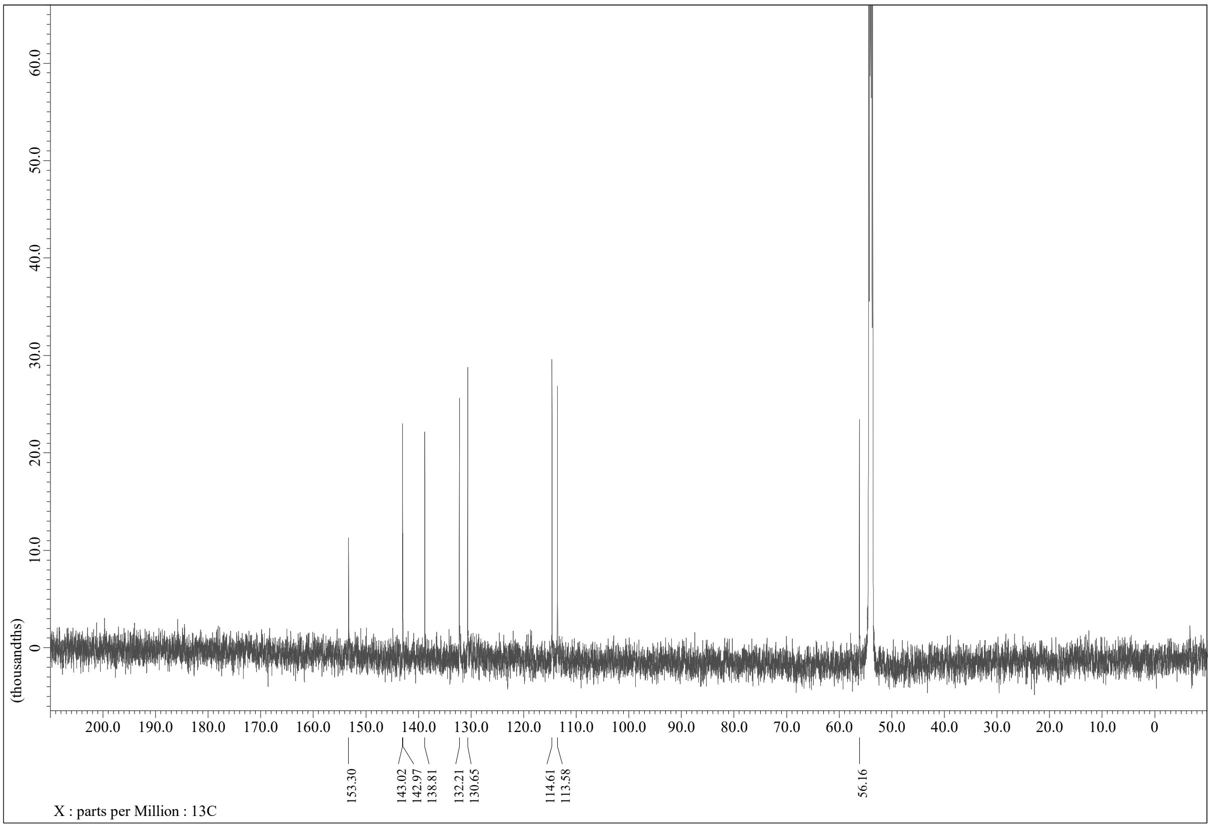

HRMS (MALDI-TOF) of **2**

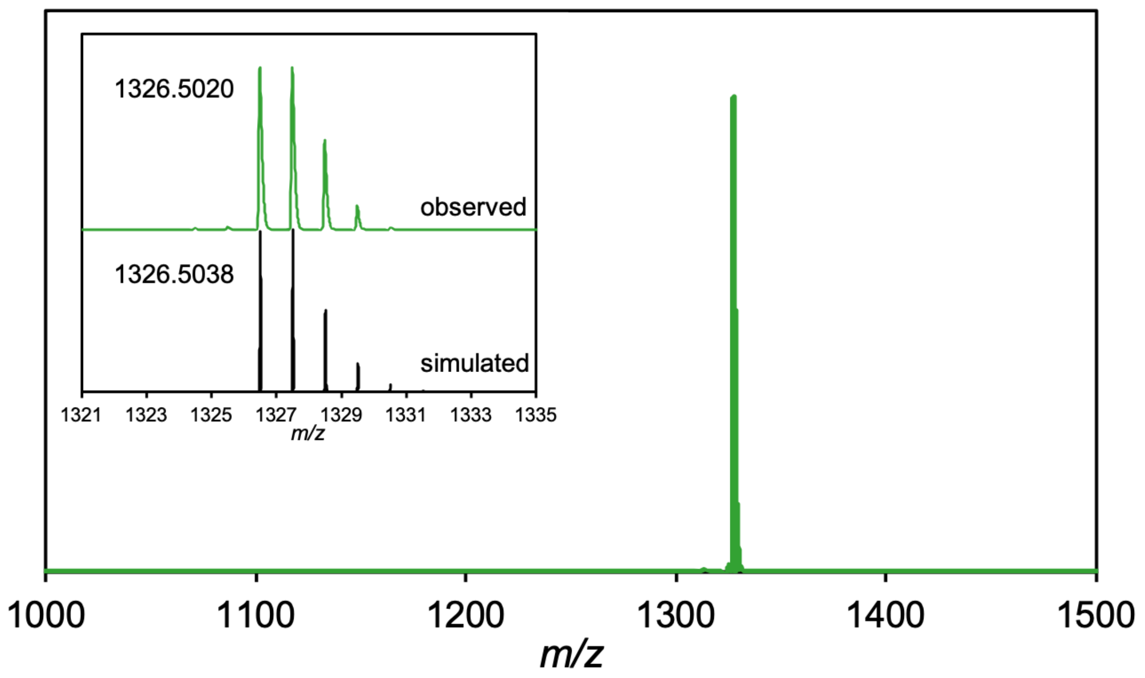

N-Belt dication $\mathbf{2}^{2+}\cdot 2[B(C_6F_5)_4]^-$

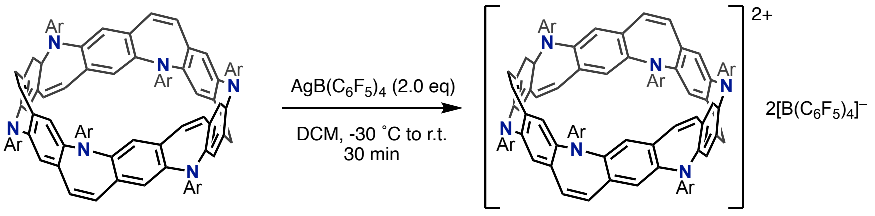

2 (Ar = *p*-methoxyphenyl) $\mathbf{2}^{2+}\cdot 2[B(C_6F_5)_4]^-$

To a dry Schlenk tube equipped with argon containing a magnetic stirring bar was added N-belt **2** (2.0 mg, 1.5 µmol). Then, dichloromethane (DCM, 1.5 mL) was added, and the mixture was cooled down to –30 °C. To the reaction mixture was slowly added 1.0 mM $Ag[B(C_6F_5)_4]$ (3.0 mL, 3.0 µmol) at –30 °C. Then, the mixture was gradually warmed up to ambient temperature and stirred for 30 min. After stirring, the mixture was concentrated under vacuum until 0.5 mL solvent remained and transferred to a glass tube through membrane filter *via* syringe. Then, the mixture was slowly diffused in hexane under argon atmosphere at –30 °C in dark. After 1 week, purple crystal appeared in the solution, which was filtered and washed by hexane under N_2 atmosphere. After dried under vacuum, N-belt dication $\mathbf{2}^{2+}\cdot 2[B(C_6F_5)_4]^-$ was obtained as a purple crystal.

3. X-ray crystallography

Details of the crystal data and a summary of the intensity data collection parameters are listed in Table S1. A suitable crystal was mounted with mineral oil on a MiTeGen MicroMounts and transferred to the goniometer of the kappa goniometer of a RIGAKU XtaLAB Synergy-S system with 1.2 kW MicroMax-007HF microfocus rotating anode (Graphite-monochromated Mo Kα radiation (λ = 0.71073 Å)) and PILATUS200K hybrid photon-counting detector. Cell parameters were determined and refined, and raw frame data were integrated using CrysAlisPro (Agilent Technologies, 2010). The structures were solved by direct methods with SHELXT[3] and refined by full-matrix least-squares techniques against F^2 (SHELXL-2018/3)[4] by using Olex2 software package.[5] The intensities were corrected for Lorentz and polarization effects. The non-hydrogen atoms were refined anisotropically. Hydrogen atoms were placed using AFIX instructions. CCDC 2133103 (**2**) and 2133104 ($\mathbf{2}^{2+}\cdot 2[B(C_6F_5)_4]^-$) contain the supplementary crystallographic data for this paper. These data can be obtained free of charge from The Cambridge Crystallographic Data Centre via www.ccdc.cam.ac.uk/data_request/cif.

Table S1. Crystallographic data for **2** and dication salt $\mathbf{2}^{2+}\cdot 2[B(C_6F_5)_4]^-$.

	**2**	$\mathbf{2}^{2+}\cdot 2[B(C_6F_5)_4]^-$
formula	C95H78N6O6	C79H55.5BCl2F20N3O3
fw	1399.63	1556.47
T (K)	123(2)	123(2)
λ (Å)	0.71073	0.71073
cryst syst	Trigonal	Monoclinic
space group	R-3	$I2/a$
a (Å)	22.2142 (14)	32.5289(11)
b (Å)	22.2142 (14)	13.8777(4)
c (Å)	12.977 (2)	32.5269(15)
α (deg)	90	90
β (deg)	90	96.618(4)
γ (deg)	120	90
V (Å^3)	5545.8 (12)	14585.7(9)
Z	3	8
D_{calc} (g / cm^3)	1.257	1.418
μ (mm^{-1})	0.079	0.1192
F(000)	2214	6348.0
cryst size (mm)	0.010 × 0.010 × 0.010	0.15 × 0.10 × 0.05
θ range (deg)	1.833–24.992	2.384–31.26
reflns collected	6084	68811
indep reflns/R_{int}	2175 / 0.0965	21919 / 0.0908
params	200	1017
GOF on F^2	0.988	1.021
R_1, wR_2 [I>2σ(I)]	0.0815, 0.1882	0.1162, 0.2728
R_1, wR_2 (all data)	0.1804, 0.2443	0.3059, 0.3935

4. Photophysical properties of N-belt 2

Absorption spectrum of 2

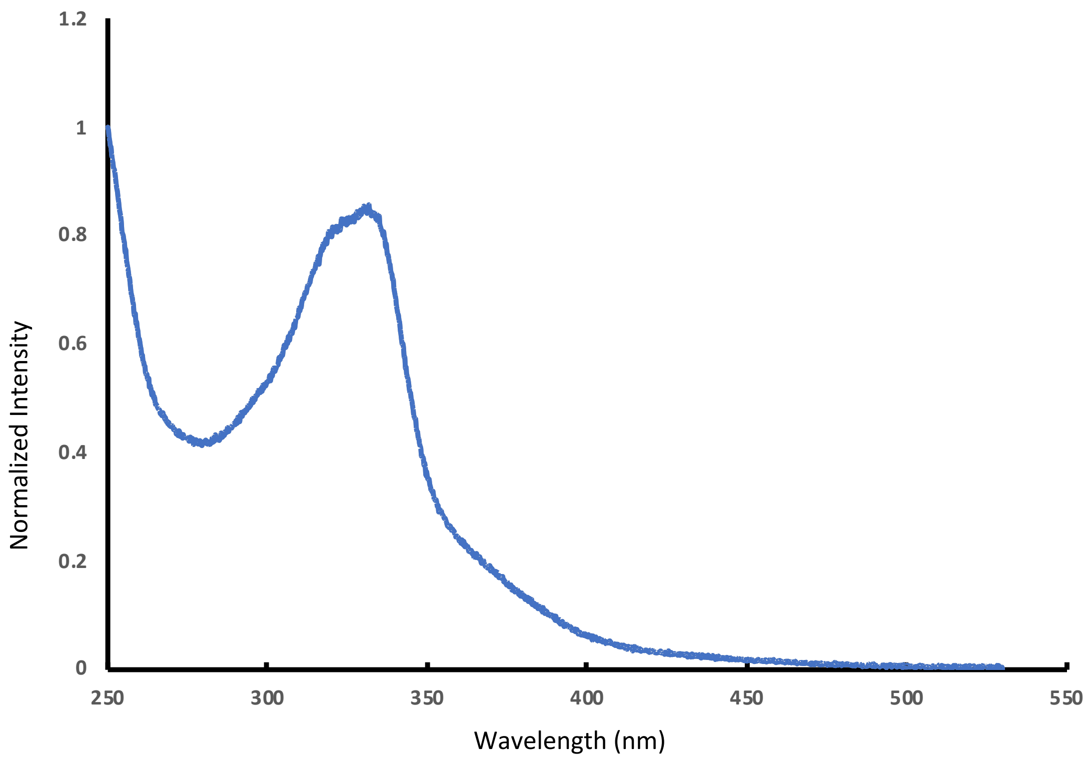

Fig S1. Absorption spectrum of **2**. Measurement was carried out under following conditions; solvent: DCM, concentration of **2**: 5.0×10^{-6} M, room temperature.

Titration of the N-belt (2) with oxidant

Dichloromethane solutions of cationic species of N-belt **2** prepared by chemical oxidation with $AgSbF_6$ were used for measurement. The samples were prepared as following the procedure: To the 0.2 mM solution of **2** in DCM was added 2.0 mM $AgSbF_6$ solution in DCM at –30 °C under argon atmosphere. Then, the reaction mixture was warmed gradually to the ambient temperature and stirred for 30 minutes at the same temperature. After stirring, the reaction mixture was diluted to 5.0×10^{-5} M in DCM and transferred to quartz cell through membrane filter by syringe.

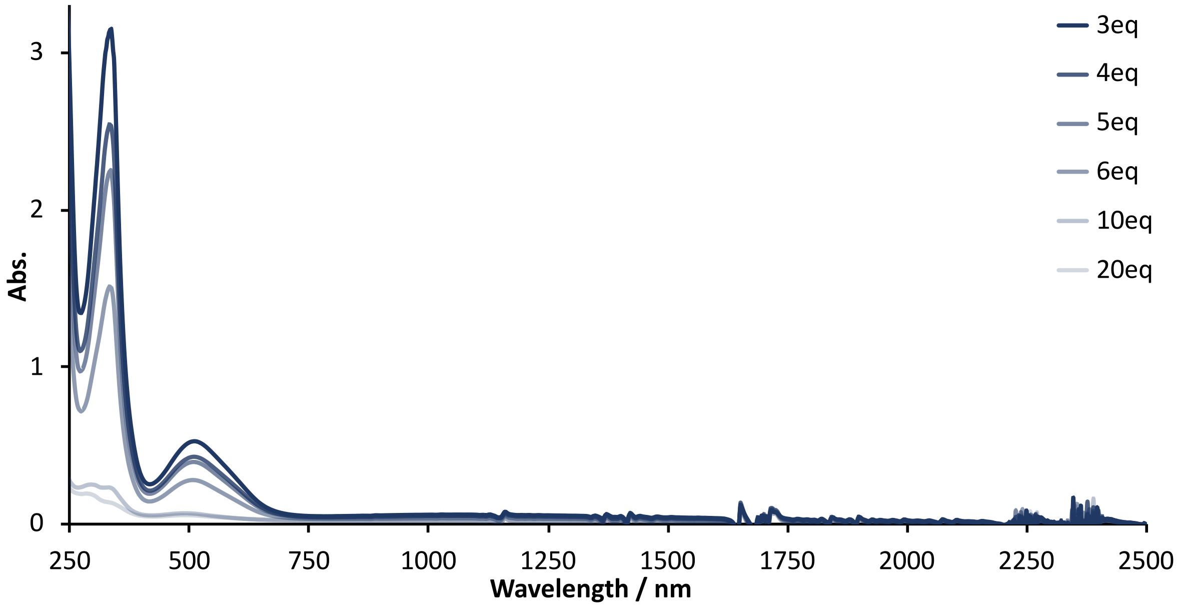

Fig S2. The UV-Vis-NIR measurement was carried out under following conditions; solvent: DCM, concentration of **2**: 5.0×10^{-5} M, oxidant: $AgSbF_6$, room temperature.

5. Electrochemical measurements of N-belt 2

Cyclic voltammetry (CV) and differential pulse voltammetry (DPV) of N-belt **2** were measured under the following conditions: 0.1 M $TBAPF_6$ and 1.0 mM **2** in DCM, Pt reference electrode, Pt working electrode, titanium counter electrode and scan rate of 100 $mV{\cdot}s^{-1}$ for CV and 20 $mV{\cdot}s^{-1}$ for DPV. The measurements were carried out under a nitrogen atmosphere.

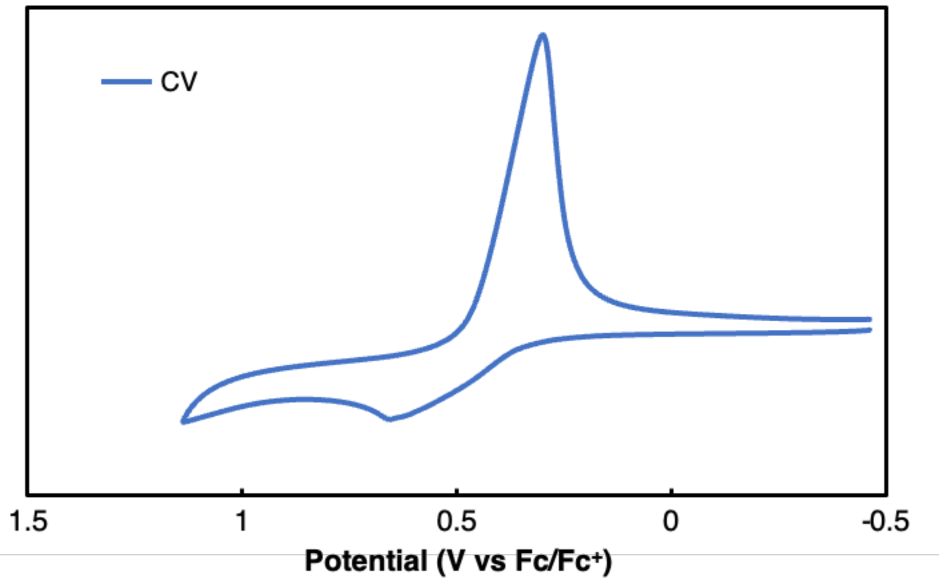

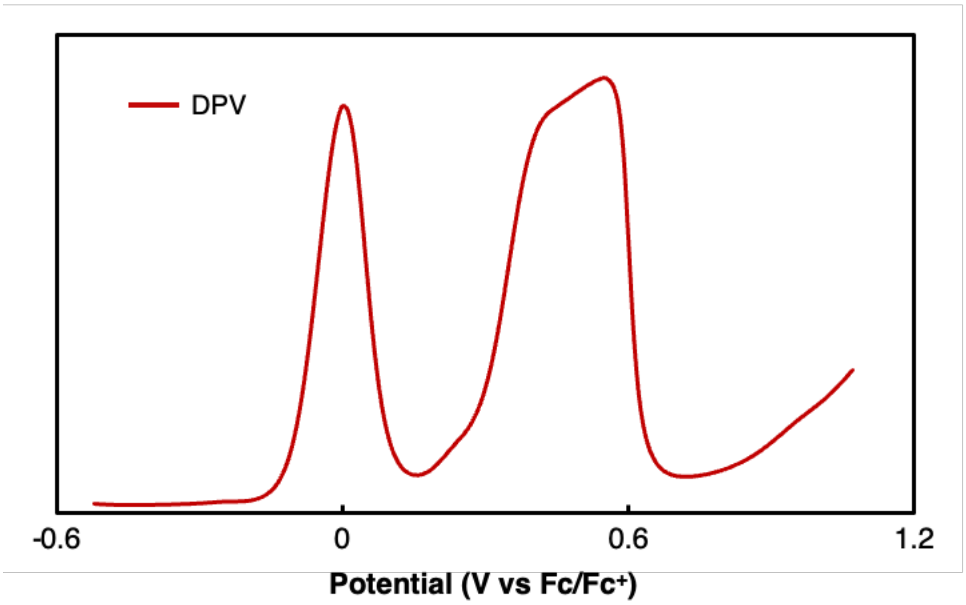

Fig S3. Cyclic voltammogram and differential pulse voltammogram of **2**. E^{Ox1} = 0.43 V (vs Fc/Fc^+), E^{Ox2} (V) = 0.54 V (vs Fc/Fc^+), where E^{Ox} were determined as the peak values in DPV.

6. Computational study

For DFT calculation, the Gaussian 16 suite of programs[6] running on a NEC LX system was used. All molecules were fully optimized using (U)B3LYP level of theory[7] with 6-31G(d) basis set. For singlet biradical states, symmetry-broken UB3LYP/6–31G(d) method was employed with a keyword “guess=mix”. Harmonic vibration frequency calculation at the same level was performed to verify all stationary points as local minima (with no imaginary frequency) or transition states (with one imaginary frequency). Time-dependent DFT (TD-DFT) calculations were performed at (U)B3LYP level of theory with 6-31G(d) basis set. Visualization of the results was performed by the use of GaussView 6.0.16 software.

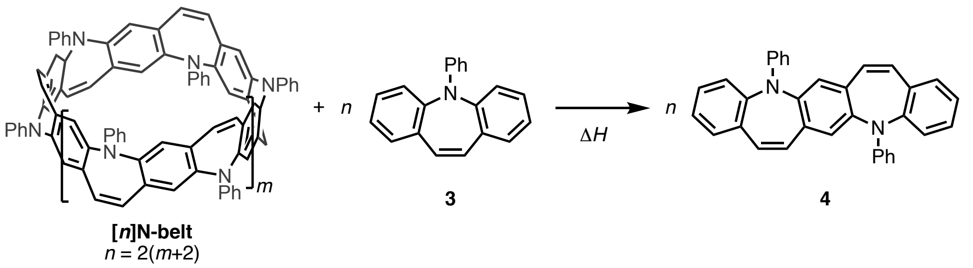

n	strain energy (kcal·mol^{-1})
4	26.0
6	1.3
8	0.4
10	9.1
12	22.7
14	39.2
16	57.6

Fig S4. (a) Hypothetical homodesmotic reactions for the calculation of the strain energies of [*n*]N-belt. (b)

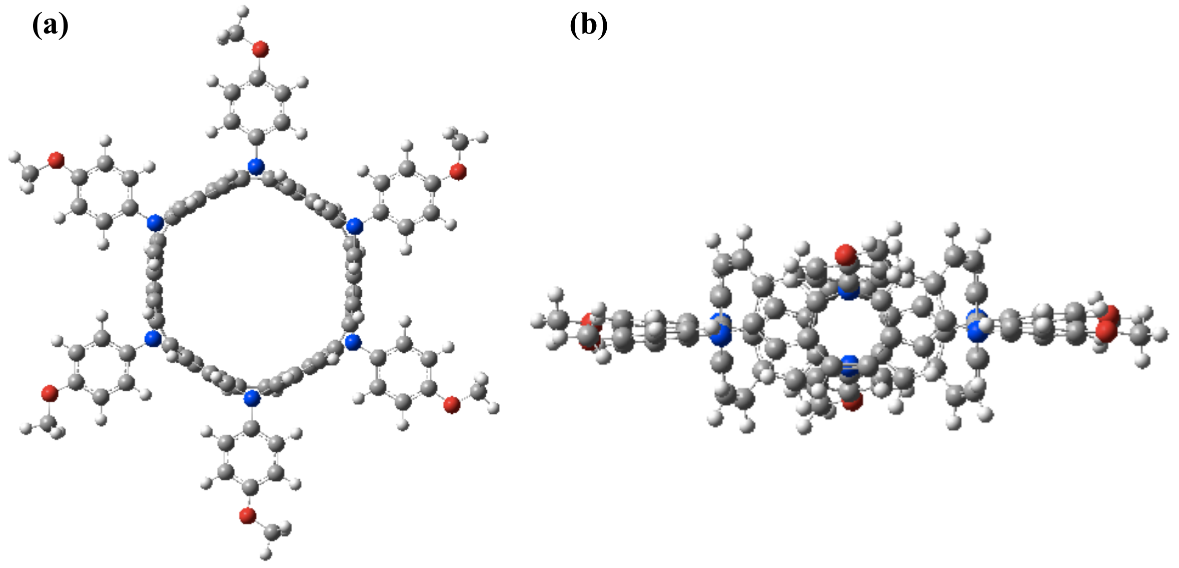

Fig S5. Optimized structure of **2**. (a) Top view; (b) side view.

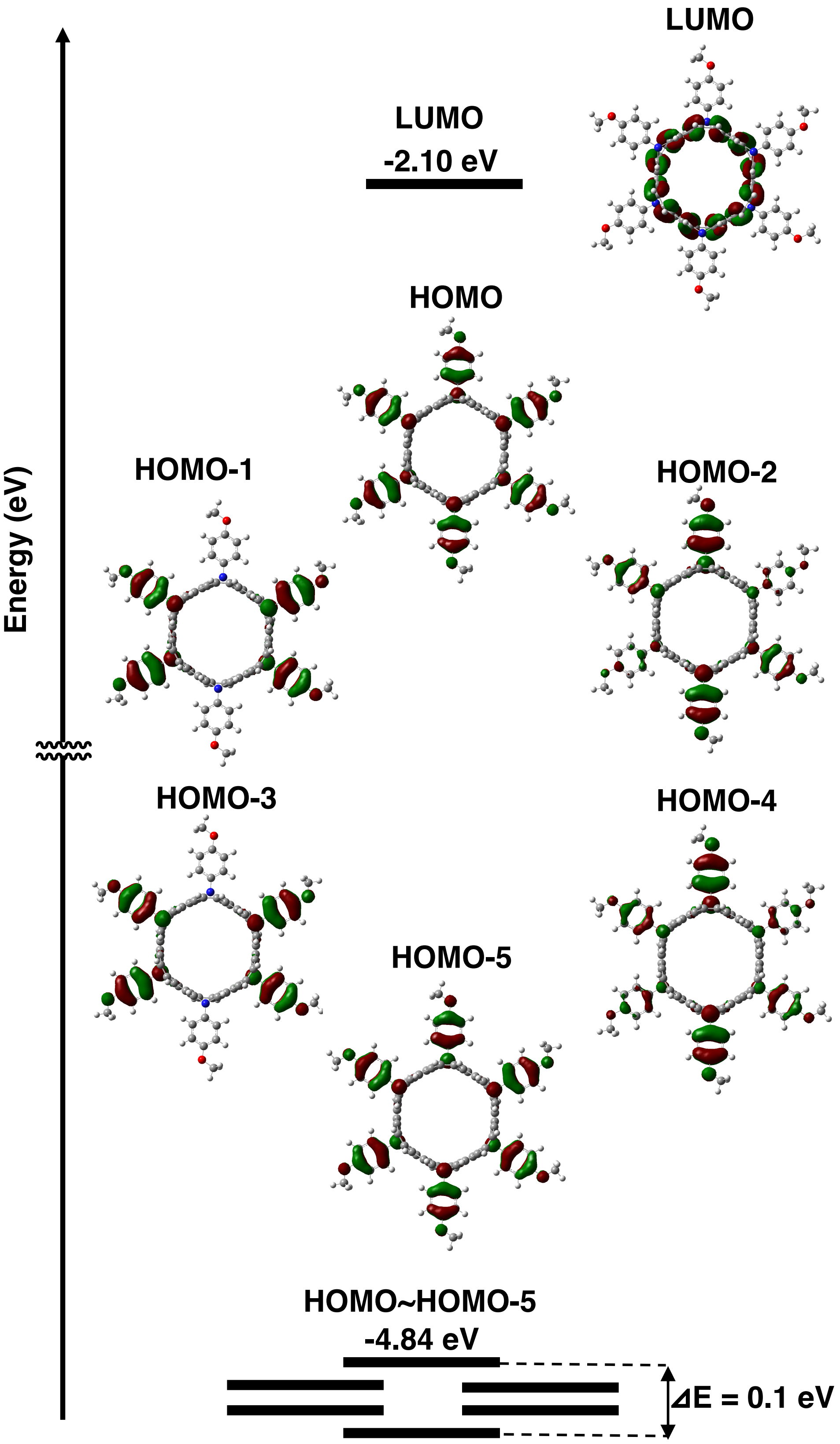

Fig S6. Frontier molecular orbitals (isovalue = 0.02) and energy diagram of **2**.

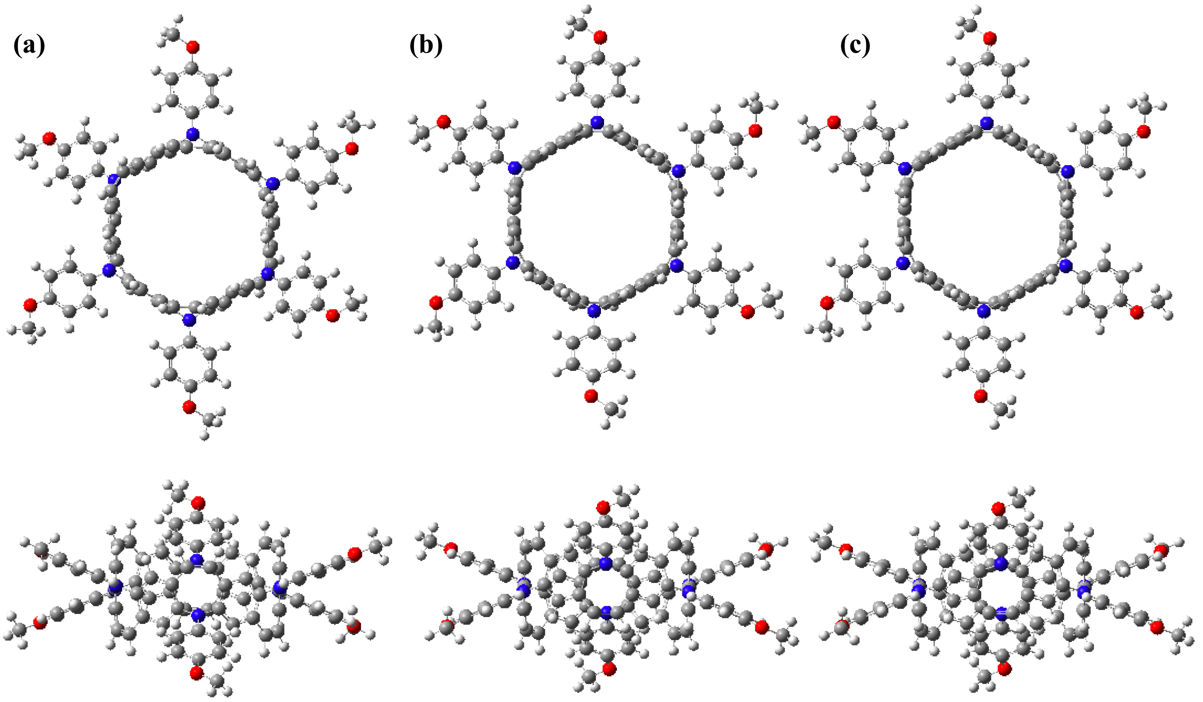

Fig S7. Optimized structure of N-belt **2** cations (a) $\mathbf{2}^{+}$ UB3LYP (b) $\mathbf{2}^{2+}$ UB3LYP_T (c) $\mathbf{2}^{3+}$ UB3LYP_Q. (T, triplet; Q, quartet).

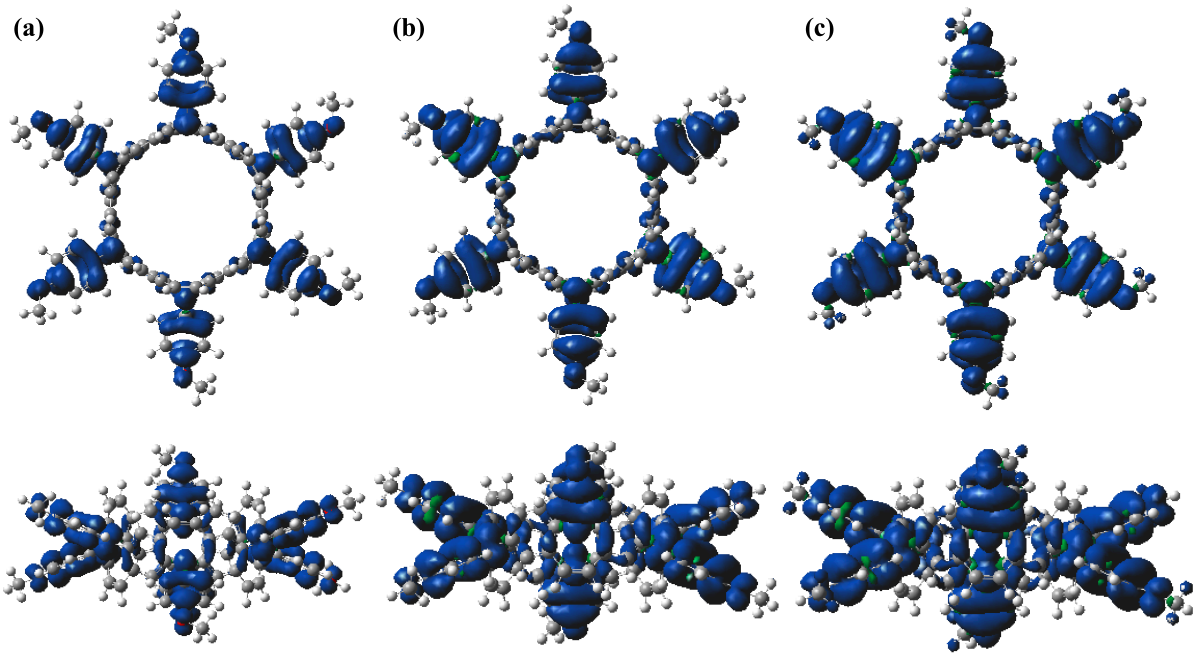

Fig S8. Spin density maps (isovalue = 0.0004) calculated for N-belt **2** cations (a) $\mathbf{2}^{+}$ UB3LYP (b) $\mathbf{2}^{2+}$ UB3LYP_T (c) $\mathbf{2}^{3+}$ UB3LYP_Q. (T, triplet; Q, quartet).

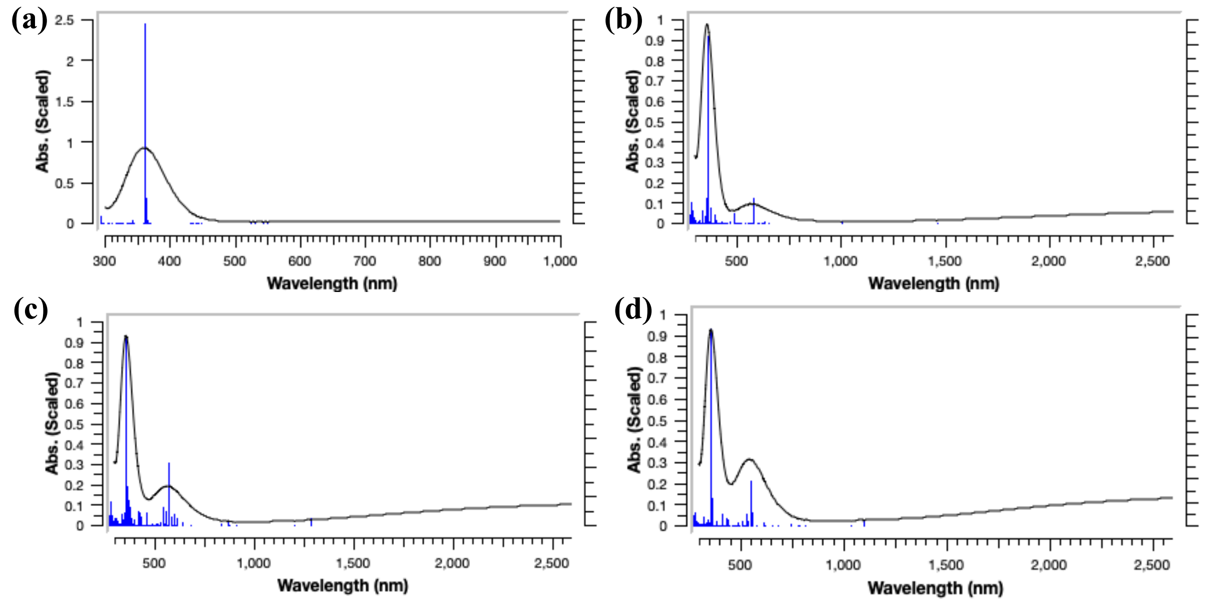

Fig S9. Time dependent-DFT (TD-DFT) calculations. Calculated stick spectra at B3LYP/6-31G(d) of (a) neutral **2** and UB3LYP/6-31G(d) of (b) radical cation $\mathbf{2}^{+}$, (c) dication $\mathbf{2}^{2+}$with triplet state, and (d) trication $\mathbf{2}^{3+}$ with quartet state.

Table S2. The TD-DFT calculation results. (a) **2** (B3LYP/6-31G(d)) (b) $\mathbf{2}^{+}$ (UB3LYP/6-31G(d)) (c) $\mathbf{2}^{2+}$ (UB3LYP/6-31G(d)_T) (d) $\mathbf{2}^{3+}$ (UB3LYP/6-31G(d)_Q)

(a) **2** (B3LYP/6-31G(d)) E = -4249.03532993 Hartree

Excited State 1: Singlet-AG 2.2523 eV 550.47 nm f=0.0000 <S**2>=0.000

346 -> 351 -0.16653

347 -> 350 0.16653

348 -> 349 0.65796

This state for optimization and/or second-order correction.

Total Energy, E(TD-HF/TD-DFT) = -4248.95256587

Copying the excited state density for this state as the 1-particle RhoCI density.

Excited State 27: Singlet-EU 3.4383 eV 360.59 nm f=0.9802 <S**2>=0.000

340 -> 349 0.18851

341 -> 349 -0.35697

342 -> 350 0.45244

342 -> 351 -0.21802

343 -> 355 -0.10423

344 -> 352 0.21848

Excited State 28: Singlet-EU 3.4383 eV 360.59 nm f=0.9803 <S**2>=0.000
340 -> 349 -0.35698
341 -> 349 -0.18851
342 -> 350 0.21802
342 -> 351 0.45244
343 -> 356 0.10423
345 -> 352 -0.21847

2^{+}(UB3LYP/6-31G(d)) E = -4248.83612625 Hartree
Excited State 1: 2.003-EU 0.2119 eV 5850.74 nm f=0.0775 <S**2>=0.753
346B -> 348B 1.19210
346B <- 348B -0.65006
This state for optimization and/or second-order correction.
Total Energy, E(TD-HF/TD-DFT) = -4248.82833863
Copying the excited state density for this state as the 1-particle RhoCI density.

Excited State 23: 2.007-EU 2.1514 eV 576.29 nm f=0.0962 <S**2>=0.757
328B -> 348B 0.20034
329B -> 348B 0.97110

Excited State 24: 2.007-EU 2.1514 eV 576.29 nm f=0.0962 <S**2>=0.757
328B -> 348B 0.97110
329B -> 348B -0.20034

Excited State 97: 2.101-EU 3.4640 eV 357.92 nm f=0.7351 <S**2>=0.854
341A -> 349A 0.14285
342A -> 350A 0.66516
343A -> 355A -0.10238
344A -> 352A -0.10233
341B -> 349B 0.22718
342B -> 350B 0.58138

Excited State 98: 2.101-EU 3.4640 eV 357.92 nm f=0.7351 <S**2>=0.854

340A -> 349A -0.14286
342A -> 351A 0.66516
343A -> 356A 0.10238
345A -> 352A 0.10233
340B -> 349B -0.22718
342B -> 351B 0.58138

2^{2+} (UB3LYP/6-31G(d)_T) E = -4248.57604166 Hartree

Excited State 1: 3.006-AG 0.0719 eV 17233.21 nm f=0.0000 <S**2>=2.010
345B -> 348B -0.28510
346B -> 347B -1.00106
346B <- 347B 0.28289
This state for optimization and/or second-order correction.
Total Energy, E(TD-HF/TD-DFT) = -4248.57339774
Copying the excited state density for this state as the 1-particle RhoCI density.

Excited State 30: 3.014-AU 2.1740 eV 570.31 nm f=0.2159 <S**2>=2.021
326B -> 347B -0.13981
327B -> 347B -0.20382
328B -> 348B 0.30062
329B -> 347B 0.39020
330B -> 348B 0.18853
331B -> 348B 0.36492
333B -> 347B 0.59224
334B -> 347B 0.18647
335B -> 347B -0.17587
336B -> 348B -0.17326
338B -> 347B 0.21487

Excited State 127: 3.260-AU 3.4726 eV 357.03 nm f=0.6545 <S**2>=2.406
340A -> 349A 0.47986
342A -> 350A 0.26839
342A -> 351A 0.18806
344A -> 357A -0.11914

340B -> 349B 0.45980
342B -> 350B 0.32007
342B -> 351B 0.17824
343B -> 354B -0.25344
343B -> 359B -0.15090
344B -> 357B 0.15959
345B -> 358B 0.16565
346B -> 355B -0.12020

Excited State 129: 3.297-AU 3.4872 eV 355.54 nm f=0.5965 <S**2>=2.468
342A -> 351A 0.57676
343A -> 355A -0.24949
344A -> 352A -0.34824
347A -> 354A 0.12599
347A -> 356A 0.10108
341B -> 349B 0.12748
342B -> 351B 0.43682
343B -> 356B 0.12317
344B -> 352B -0.12182

2^{3+} (UB3LYP/6-31G(d)_Q) E = -4248.25556306 Hartree
Excited State 1: 4.009-AU 0.0993 eV 12490.76 nm f=0.0000 <S**2>=3.767
344B -> 346B -0.73678
344B -> 347B -0.11675
345B -> 346B -0.11663
345B -> 347B 0.73733
344B <- 346B -0.23636
345B <- 347B 0.23584
This state for optimization and/or second-order correction.
Total Energy, E(TD-HF/TD-DFT) = -4248.25191530
Copying the excited state density for this state as the 1-particle RhoCI density

Excited State 138: 4.051-AU 3.4635 eV 357.97 nm f=0.8372 <S**2>=3.854
341A -> 349A 0.25796

342A -> 350A 0.64187

341B -> 349B 0.34661

342B -> 350B 0.55996

Excited State 139: 4.051-AU 3.4637 eV 357.95 nm f=0.8356 <S**2>=3.854

340A -> 349A -0.25770

342A -> 351A 0.64207

340B -> 349B -0.34691

342B -> 351B 0.55980

Table S3. Cartesian coordinates of optimized structures with uncorrected and thermal-corrected (298 K) energies of stationary points (Hartree).

[4]N-belt E = -2374.56844998, H = -2373.764196

C	-3.330151	0.674356	2.173844
C	-3.330151	-0.674356	2.173844
C	-2.820858	-1.491819	1.061317
C	-3.113525	-1.172321	-0.282794
N	-3.898030	0.000000	-0.542259
C	-3.113525	1.172321	-0.282794
C	-2.820858	1.491819	1.061317
C	-2.493364	1.873597	-1.318569
C	-1.491819	2.820858	-1.061317
C	-1.172321	3.113525	0.282794
C	-1.873597	2.493364	1.318569
C	-1.873597	-2.493364	1.318569
C	-1.172321	-3.113525	0.282794
C	-1.491819	-2.820858	-1.061317
C	-2.493364	-1.873597	-1.318569
C	-5.058943	0.000000	-1.330819
C	-5.672937	1.209086	-1.710738
C	-6.846483	1.199968	-2.461287
C	-7.443823	0.000000	-2.849998
C	-6.846483	-1.199968	-2.461287
C	-5.672937	-1.209086	-1.710738
C	-1.209086	-5.672937	1.710738
C	-1.199968	-6.846483	2.461287
C	0.000000	-7.443823	2.849998
C	1.199968	-6.846483	2.461287
C	1.209086	-5.672937	1.710738
C	0.000000	-5.058943	1.330819
N	0.000000	-3.898030	0.542259
C	0.674356	-3.330151	-2.173844
C	-0.674356	-3.330151	-2.173844
C	3.330151	-0.674356	2.173844
C	3.330151	0.674356	2.173844
C	2.820858	1.491819	1.061317
C	3.113525	1.172321	-0.282794
N	3.898030	0.000000	-0.542259
C	1.873597	2.493364	1.318569
C	1.172321	3.113525	0.282794
C	1.491819	2.820858	-1.061317
C	2.493364	1.873597	-1.318569
C	5.058943	0.000000	-1.330819
C	5.672937	-1.209086	-1.710738
C	6.846483	-1.199968	-2.461287
C	7.443823	0.000000	-2.849998
C	6.846483	1.199968	-2.461287
C	5.672937	1.209086	-1.710738
C	-1.209086	5.672937	1.710738
C	-1.199968	6.846483	2.461287
C	0.000000	7.443823	2.849998
C	1.199968	6.846483	2.461287
C	1.209086	5.672937	1.710738
C	0.000000	5.058943	1.330819
N	0.000000	3.898030	0.542259
C	-0.674356	3.330151	-2.173844
C	0.674356	3.330151	-2.173844
C	3.113525	-1.172321	-0.282794
C	2.820858	-1.491819	1.061317
C	2.493364	-1.873597	-1.318569
C	1.491819	-2.820858	-1.061317
C	1.172321	-3.113525	0.282794
C	1.873597	-2.493364	1.318569
H	-3.593441	1.198258	3.091102
H	-3.593441	-1.198258	3.091102
H	-2.718614	1.600789	-2.345708
H	-1.600789	2.718614	2.345708
H	-1.600789	-2.718614	2.345708
H	-2.718614	-1.600789	-2.345708
H	-5.236592	2.154227	-1.407034
H	-7.298431	2.148847	-2.739669
H	-8.357416	0.000000	-3.437014
H	-7.298431	-2.148847	-2.739669
H	-5.236592	-2.154227	-1.407034
H	-2.154227	-5.236592	1.407034
H	-2.148847	-7.298431	2.739669
H	0.000000	-8.357416	3.437014
H	2.148847	-7.298431	2.739669
H	2.154227	-5.236592	1.407034
H	1.198258	-3.593441	-3.091102
H	-1.198258	-3.593441	-3.091102
H	3.593441	-1.198258	3.091102
H	3.593441	1.198258	3.091102
H	1.600789	2.718614	2.345708
H	2.718614	1.600789	-2.345708
H	5.236592	-2.154227	-1.407034
H	7.298431	-2.148847	-2.739669
H	8.357416	0.000000	-3.437014
H	7.298431	2.148847	-2.739669
H	5.236592	2.154227	-1.407034
H	-2.154227	5.236592	1.407034
H	-2.148847	7.298431	2.739669
H	0.000000	8.357416	3.437014
H	2.148847	7.298431	2.739669
H	2.154227	5.236592	1.407034
H	-1.198258	3.593441	-3.091102
H	1.198258	3.593441	-3.091102
H	2.718614	-1.600789	-2.345708
H	1.600789	-2.718614	2.345708

[6]N-belt E = -3561.91532987, H = -3560.706386

C	1.202119	4.822237	0.298147
C	1.548579	4.594865	-1.054406
C	2.024040	4.352751	1.319349
C	3.204980	3.638541	1.054406
C	3.575120	3.452184	-0.298147
C	2.757573	3.929245	-1.319349
C	-4.639439	-1.897489	-2.166328
C	-3.962993	-3.069127	-2.166328
C	-3.204980	-3.638541	-1.054406
C	-3.575120	-3.452184	0.298147
N	-4.785659	-2.763001	0.624767
C	-4.777240	-1.370053	0.298147
C	-4.753559	-0.956324	-1.054406
C	-4.781613	-0.423506	1.319349
C	-4.753559	0.956324	1.054406
C	-4.777240	1.370053	-0.298147
C	-4.781613	0.423506	-1.319349
C	-2.024040	-4.352751	-1.319349
C	-1.202119	-4.822237	-0.298147
C	-1.548579	-4.594865	1.054406
C	-2.757573	-3.929245	1.319349
C	-5.997489	-3.462652	0.815662
C	-7.218893	-2.772339	0.925649
C	-8.406255	-3.468220	1.143438
C	-8.416007	-4.858984	1.255899
C	-7.206694	-5.545921	1.143438
C	-6.010363	-4.865575	0.925649
C	-1.208530	-7.637914	-0.925649
C	-1.199561	-9.014140	-1.143438
C	0.000000	-9.717967	-1.255899
C	0.000000	9.717967	1.255899
C	-1.199561	9.014140	1.143438
C	-1.208530	7.637914	0.925649
C	-7.218893	2.772339	-0.925649
C	-8.406255	3.468220	-1.143438
C	-8.416007	4.858984	-1.255899
C	-7.206694	5.545921	-1.143438
C	-6.010363	4.865575	-0.925649
C	-5.997489	3.462652	-0.815662
N	-4.785659	2.763001	-0.624767
C	-4.639439	1.897489	2.166328
C	-3.962993	3.069127	2.166328
C	3.962993	-3.069127	-2.166328
C	4.639439	-1.897489	-2.166328
C	4.753559	-0.956324	-1.054406
C	4.777240	-1.370053	0.298147
N	4.785659	-2.763001	0.624767
C	3.575120	-3.452184	0.298147
C	3.204980	-3.638541	-1.054406
C	2.757573	-3.929245	1.319349
C	1.548579	-4.594865	1.054406
C	1.202119	-4.822237	-0.298147
C	2.024040	-4.352751	-1.319349
C	4.781613	0.423506	-1.319349
C	4.777240	1.370053	-0.298147
C	4.753559	0.956324	1.054406
C	4.781613	-0.423506	1.319349
C	5.997489	-3.462652	0.815662
C	7.218893	-2.772339	0.925649
C	8.406255	-3.468220	1.143438
H	-1.720440	-4.529000	-2.347426
H	-3.062009	-3.754445	2.347426
H	-7.240060	-1.691886	0.838921
H	-9.335000	-2.908503	1.223137
H	-9.344891	-5.395275	1.425934
H	-7.186337	-6.630096	1.223137
H	-5.085247	-5.424133	0.838921
H	-2.154813	-7.116019	-0.838921
H	-2.148663	-9.538598	-1.223137
H	0.000000	-10.790551	-1.425934
H	2.148663	-9.538598	-1.223137
H	2.154813	-7.116019	-0.838921
H	1.177052	-5.147030	3.115878
H	-1.177052	-5.147030	3.115878
H	1.177052	5.147030	-3.115878
H	-1.177052	5.147030	-3.115878
H	-3.062009	3.754445	-2.347426
H	-1.720440	4.529000	2.347426
H	2.154813	7.116019	0.838921
H	2.148663	9.538598	1.223137
H	0.000000	10.790551	1.425934
H	-2.148663	9.538598	1.223137
H	-2.154813	7.116019	0.838921
H	-7.240060	1.691886	-0.838921
H	-9.335000	2.908503	-1.223137
H	-9.344891	5.395275	-1.425934
H	-7.186337	6.630096	-1.223137
H	-5.085247	5.424133	-0.838921
H	-5.045985	1.554158	3.115878
H	-3.868933	3.592872	3.115878

C	1.199561	-9.014140	-1.143438
C	1.208530	-7.637914	-0.925649
C	0.000000	-6.925304	-0.815662
N	0.000000	-5.526003	-0.624767
C	0.676445	-4.966616	2.166328
C	-0.676445	-4.966616	2.166328
C	0.676445	4.966616	-2.166328
C	-0.676445	4.966616	-2.166328
C	-1.548579	4.594865	-1.054406
C	-1.202119	4.822237	0.298147
N	0.000000	5.526003	0.624767
C	-2.757573	3.929245	-1.319349
C	-3.575120	3.452184	-0.298147
C	-3.204980	3.638541	1.054406
C	-2.024040	4.352751	1.319349
C	0.000000	6.925304	0.815662
C	1.208530	7.637914	0.925649
C	1.199561	9.014140	1.143438
C	8.416007	-4.858984	1.255899
C	7.206694	-5.545921	1.143438
C	6.010363	-4.865575	0.925649
C	6.010363	4.865575	-0.925649
C	7.206694	5.545921	-1.143438
C	8.416007	4.858984	-1.255899
C	8.406255	3.468220	-1.143438
C	7.218893	2.772339	-0.925649
C	5.997489	3.462652	-0.815662
N	4.785659	2.763001	-0.624767
C	3.962993	3.069127	2.166328
C	4.639439	1.897489	2.166328
H	1.720440	4.529000	2.347426
H	3.062009	3.754445	-2.347426
H	-5.045985	-1.554158	-3.115878
H	-3.868933	-3.592872	-3.115878
H	-4.782449	-0.774555	2.347426
H	-4.782449	0.774555	-2.347426
H	3.868933	-3.592872	-3.115878
H	5.045985	-1.554158	-3.115878
H	3.062009	-3.754445	2.347426
H	1.720440	-4.529000	-2.347426
H	4.782449	0.774555	-2.347426
H	4.782449	-0.774555	2.347426
H	7.240060	-1.691886	0.838921
H	9.335000	-2.908503	1.223137
H	9.344891	-5.395275	1.425934
H	7.186337	-6.630096	1.223137
H	5.085247	-5.424133	0.838921
H	5.085247	5.424133	-0.838921
H	7.186337	6.630096	-1.223137
H	9.344891	5.395275	-1.425934
H	9.335000	2.908503	-1.223137
H	7.240060	1.691886	-0.838921
H	3.868933	3.592872	3.115878
H	5.045985	1.554158	3.115878

[8]N-belt E = -4749.22439494, H = -4747.610812

C	1.215866	6.513821	0.307434
C	1.570750	6.316777	-1.048365
C	2.086049	6.118094	1.319097
C	3.355948	5.577324	1.048365
C	3.746219	5.465714	-0.307434
C	2.851086	5.801205	-1.319097
C	-6.617992	0.677055	-2.159946
C	-6.617992	-0.677055	-2.159946
C	-6.316777	-1.570750	-1.048365
C	-6.513821	-1.215866	0.307434
N	-7.181509	0.000000	0.652099
C	-6.513821	1.215866	0.307434
C	-6.316777	1.570750	-1.048365
C	-6.118094	2.086049	1.319097
C	-5.577324	3.355948	1.048365
C	-5.465714	3.746219	-0.307434
C	-5.801205	2.851086	-1.319097
C	-5.801205	-2.851086	-1.319097
C	-5.465714	-3.746219	-0.307434
C	-5.577324	-3.355948	1.048365
C	-6.118094	-2.086049	1.319097
C	-8.576585	0.000000	0.902931
C	-9.287807	1.207459	1.038208
C	-10.655153	1.198542	1.306923
C	-11.355038	0.000000	1.446285
C	-10.655153	-1.198542	1.306923
C	-9.287807	-1.207459	1.038208
C	-7.421274	-5.713669	-1.038208
C	-8.381828	-6.686834	-1.306923
C	-8.029225	-8.029225	-1.446285
C	-6.686834	-8.381828	-1.306923
C	-5.713669	-7.421274	-1.038208
C	-6.064561	-6.064561	-0.902931
N	-5.078093	-5.078093	-0.652099
C	-4.200877	-5.158378	2.159946
C	-5.158378	-4.200877	2.159946
C	0.677055	6.617992	-2.159946
C	-0.677055	6.617992	-2.159946
C	-1.570750	6.316777	-1.048365
C	-1.215866	6.513821	0.307434
N	0.000000	7.181509	0.652099
C	-2.851086	5.801205	-1.319097
C	-3.746219	5.465714	-0.307434
C	-3.355948	5.577324	1.048365
C	-2.086049	6.118094	1.319097
C	0.000000	8.576585	0.902931
C	1.207459	9.287807	1.038208
C	1.198542	10.655153	1.306923
C	0.000000	11.355038	1.446285
C	6.316777	-1.570750	-1.048365
C	6.118094	-2.086049	1.319097
C	5.577324	-3.355948	1.048365
C	5.465714	-3.746219	-0.307434
C	5.801205	-2.851086	-1.319097
C	8.576585	0.000000	0.902931
C	9.287807	-1.207459	1.038208
C	10.655153	-1.198542	1.306923
C	11.355038	0.000000	1.446285
C	10.655153	1.198542	1.306923
C	9.287807	1.207459	1.038208
C	-0.677055	-6.617992	-2.159946
C	0.677055	-6.617992	-2.159946
C	1.570750	-6.316777	-1.048365
C	1.215866	-6.513821	0.307434
N	0.000000	-7.181509	0.652099
C	-1.215866	-6.513821	0.307434
C	-1.570750	-6.316777	-1.048365
C	-2.086049	-6.118094	1.319097
C	-3.355948	-5.577324	1.048365
C	-3.746219	-5.465714	-0.307434
C	-2.851086	-5.801205	-1.319097
C	2.851086	-5.801205	-1.319097
C	3.746219	-5.465714	-0.307434
C	3.355948	-5.577324	1.048365
C	2.086049	-6.118094	1.319097
C	0.000000	-8.576585	0.902931
C	1.207459	-9.287807	1.038208
C	1.198542	-10.655153	1.306923
C	0.000000	-11.355038	1.446285
C	-1.198542	-10.655153	1.306923
C	-1.207459	-9.287807	1.038208
C	7.421274	-5.713669	-1.038208
C	8.381828	-6.686834	-1.306923
C	8.029225	-8.029225	-1.446285
C	6.686834	-8.381828	-1.306923
C	5.713669	-7.421274	-1.038208
C	6.064561	-6.064561	-0.902931
N	5.078093	-5.078093	-0.652099
C	5.158378	-4.200877	2.159946
C	4.200877	-5.158378	2.159946
C	6.316777	1.570750	-1.048365
C	6.513821	1.215866	0.307434
C	5.801205	2.851086	-1.319097
C	5.465714	3.746219	-0.307434
C	5.577324	3.355948	1.048365
C	6.118094	2.086049	1.319097
C	5.713669	7.421274	-1.038208
C	6.686834	8.381828	-1.306923
H	-8.774776	2.155896	0.932060
H	-11.175173	2.148341	1.405051
H	-12.420451	0.000000	1.656572
H	-11.175173	-2.148341	1.405051
H	-8.774776	-2.155896	0.932060
H	-7.729152	-4.680255	-0.932060
H	-9.421147	-6.382934	-1.405051
H	-8.782585	-8.782585	-1.656572
H	-6.382934	-9.421147	-1.405051
H	-4.680255	-7.729152	-0.932060
H	-3.954716	-5.607767	3.120274
H	-5.607767	-3.954716	3.120274
H	1.168884	6.761696	-3.120274
H	-1.168884	6.761696	-3.120274
H	-3.173620	5.684120	-2.349865
H	-1.775192	6.263368	2.349865
H	2.155896	8.774776	0.932060
H	2.148341	11.175173	1.405051
H	0.000000	12.420451	1.656572
H	-2.148341	11.175173	1.405051
H	-2.155896	8.774776	0.932060
H	-7.729152	4.680255	-0.932060
H	-9.421147	6.382934	-1.405051
H	-8.782585	8.782585	-1.656572
H	-6.382934	9.421147	-1.405051
H	-4.680255	7.729152	-0.932060
H	-5.607767	3.954716	3.120274
H	-3.954716	5.607767	3.120274
H	6.761696	-1.168884	-3.120274
H	6.761696	1.168884	-3.120274
H	6.263368	-1.775192	2.349865
H	5.684120	-3.173620	-2.349865
H	8.774776	-2.155896	0.932060
H	11.175173	-2.148341	1.405051
H	12.420451	0.000000	1.656572
H	11.175173	2.148341	1.405051
H	8.774776	2.155896	0.932060
H	-1.168884	-6.761696	-3.120274
H	1.168884	-6.761696	-3.120274
H	-1.775192	-6.263368	2.349865
H	-3.173620	-5.684120	-2.349865
H	3.173620	-5.684120	-2.349865
H	1.775192	-6.263368	2.349865
H	2.155896	-8.774776	0.932060
H	2.148341	-11.175173	1.405051
H	0.000000	-12.420451	1.656572
H	-2.148341	-11.175173	1.405051
H	-2.155896	-8.774776	0.932060
H	7.729152	-4.680255	-0.932060

C	-1.198542	10.655153	1.306923
C	-1.207459	9.287807	1.038208
C	-7.421274	5.713669	-1.038208
C	-8.381828	6.686834	-1.306923
C	-8.029225	8.029225	-1.446285
C	-6.686834	8.381828	-1.306923
C	-5.713669	7.421274	-1.038208
C	-6.064561	6.064561	-0.902931
N	-5.078093	5.078093	-0.652099
C	-5.158378	4.200877	2.159946
C	-4.200877	5.158378	2.159946
C	6.617992	-0.677055	-2.159946
C	6.617992	0.677055	-2.159946
N	7.181509	0.000000	0.652099
C	6.513821	-1.215866	0.307434
C	8.029225	8.029225	-1.446285
C	8.381828	6.686834	-1.306923
C	7.421274	5.713669	-1.038208
C	6.064561	6.064561	-0.902931
N	5.078093	5.078093	-0.652099
C	4.200877	5.158378	2.159946
C	5.158378	4.200877	2.159946
H	1.775192	6.263368	2.349865
H	3.173620	5.684120	-2.349865
H	-6.761696	1.168884	-3.120274
H	-6.761696	-1.168884	-3.120274
H	-6.263368	1.775192	2.349865
H	-5.684120	3.173620	-2.349865
H	-5.684120	-3.173620	-2.349865
H	-6.263368	-1.775192	2.349865
H	9.421147	-6.382934	-1.405051
H	8.782585	-8.782585	-1.656572
H	6.382934	-9.421147	-1.405051
H	4.680255	-7.729152	-0.932060
H	5.607767	-3.954716	3.120274
H	3.954716	-5.607767	3.120274
H	5.684120	3.173620	-2.349865
H	6.263368	1.775192	2.349865
H	4.680255	7.729152	-0.932060
H	6.382934	9.421147	-1.405051
H	8.782585	8.782585	-1.656572
H	9.421147	6.382934	-1.405051
H	7.729152	4.680255	-0.932060
H	3.954716	5.607767	3.120274
H	5.607767	3.954716	3.120274

[10]N-belt E = -5936.51782754, H = -5934.499792

C	-1.223383	8.194358	0.312046
C	-1.582282	8.012997	-1.045322
C	-2.118410	7.844548	1.318515
C	-3.429828	7.412693	1.045322
C	-3.826785	7.348462	-0.312046
C	-2.897080	7.591542	-1.318515
C	7.652534	3.198729	-2.157403
C	8.071196	1.910220	-2.157403
C	8.109765	0.971312	-1.045322
C	8.171344	1.368690	0.312046
N	8.409254	2.732332	0.668035
C	7.415252	3.695702	0.312046
C	7.131861	3.980992	-1.045322
C	6.805984	4.438826	1.318515
C	5.990015	5.552608	1.045322
C	5.806261	5.910289	-0.312046
C	6.324739	5.101202	-1.318515
C	8.115233	-0.409371	-1.318515
C	8.171344	-1.368690	-0.312046
C	8.109765	-0.971312	1.045322
C	8.115233	0.409371	1.318515
C	9.741172	3.165099	0.912790
C	10.046905	4.533365	1.044077
C	11.351744	4.948018	1.304479
C	12.388778	4.025358	1.439512
C	12.092126	2.669357	1.304479
C	10.792762	2.237854	1.044077
C	10.792762	-2.237854	-1.044077
C	12.092126	-2.669357	-1.304479
C	12.388778	-4.025358	-1.439512
C	11.351744	-4.948018	-1.304479
C	10.046905	-4.533365	-1.044077
C	9.741172	-3.165099	-0.912790
N	8.409254	-2.732332	-0.668035
C	7.652534	-3.198729	2.157403
C	8.071196	-1.910220	2.157403
C	-0.677409	8.266454	-2.157403
C	0.677409	8.266454	-2.157403
C	1.582282	8.012997	-1.045322
C	1.223383	8.194358	0.312046
N	0.000000	8.842012	0.668035
C	2.897080	7.591542	-1.318515
C	3.826785	7.348462	-0.312046
C	3.429828	7.412693	1.045322
C	2.118410	7.844548	1.318515
C	0.000000	10.242474	0.912790
C	-1.206822	10.956062	1.044077
C	-1.197963	12.325172	1.304479
C	0.000000	13.026332	1.439512
C	1.197963	12.325172	1.304479
C	1.206822	10.956062	1.044077
C	7.416151	8.154288	-1.044077
C	5.806261	-5.910289	0.312046
C	5.990015	-5.552608	-1.045322
C	6.324739	-5.101202	1.318515
C	7.131861	-3.980992	1.045322
C	7.415252	-3.695702	-0.312046
C	6.805984	-4.438826	-1.318515
C	2.118410	-7.844548	-1.318515
C	1.223383	-8.194358	-0.312046
C	1.582282	-8.012997	1.045322
C	2.897080	-7.591542	1.318515
C	6.020375	-8.286336	0.912790
C	5.463472	-9.572992	1.044077
C	6.275381	-10.675418	1.304479
C	7.656686	-10.538524	1.439512
C	8.213727	-9.267128	1.304479
C	7.416151	-8.154288	1.044077
C	-1.206822	-10.956062	-1.044077
C	-1.197963	-12.325172	-1.304479
C	0.000000	-13.026332	-1.439512
C	1.197963	-12.325172	-1.304479
C	1.206822	-10.956062	-1.044077
C	0.000000	-10.242474	-0.912790
N	0.000000	-8.842012	-0.668035
C	-0.677409	-8.266454	2.157403
C	0.677409	-8.266454	2.157403
C	-8.071196	1.910220	-2.157403
C	-7.652534	3.198729	-2.157403
C	-7.131861	3.980992	-1.045322
C	-7.415252	3.695702	0.312046
N	-8.409254	2.732332	0.668035
C	-8.171344	1.368690	0.312046
C	-8.109765	0.971312	-1.045322
C	-8.115233	0.409371	1.318515
C	-8.109765	-0.971312	1.045322
C	-8.171344	-1.368690	-0.312046
C	-8.115233	-0.409371	-1.318515
C	-6.324739	5.101202	-1.318515
C	-5.806261	5.910289	-0.312046
C	-5.990015	5.552608	1.045322
C	-6.805984	4.438826	1.318515
C	-9.741172	3.165099	0.912790
C	-10.792762	2.237854	1.044077
C	-12.092126	2.669357	1.304479
C	-12.388778	4.025358	1.439512
C	-11.351744	4.948018	1.304479
C	-10.046905	4.533365	1.044077
C	-5.463472	9.572992	-1.044077
C	-6.275381	10.675418	-1.304479
C	-7.656686	10.538524	-1.439512
C	-8.213727	9.267128	-1.304479
C	-7.416151	8.154288	-1.044077
C	-6.020375	8.286336	-0.912790
H	13.403245	4.354978	1.643344
H	12.880067	1.926265	1.399811
H	10.600511	1.176673	0.943644
H	10.600511	-1.176673	-0.943644
H	12.880067	-1.926265	-1.399811
H	13.403245	-4.354978	-1.643344
H	11.552423	-6.012332	-1.399811
H	9.267624	-5.278875	-0.943644
H	7.614754	-3.698714	3.123479
H	8.334515	-1.483518	3.123479
H	-1.164597	8.385027	-3.123479
H	1.164597	8.385027	-3.123479
H	3.226268	7.508628	-2.350508
H	1.803355	7.970961	2.350508
H	-2.156655	10.445297	0.943644
H	-2.148172	12.844920	1.399811
H	0.000000	14.093006	1.643344
H	2.148172	12.844920	1.399811
H	2.156655	10.445297	0.943644
H	7.884362	7.182772	-0.943644
H	9.287963	9.129095	-1.399811
H	8.283661	11.401481	-1.643344
H	5.812147	11.654423	-1.399811
H	4.394821	9.718073	-0.943644
H	5.870774	6.099096	3.123479
H	3.986416	7.468162	3.123479
H	-3.986416	-7.468162	-3.123479
H	-5.870774	-6.099096	-3.123479
H	-3.226268	-7.508628	2.350508
H	-1.803355	-7.970961	-2.350508
H	-4.394821	-9.718073	0.943644
H	-5.812147	-11.654423	1.399811
H	-8.283661	-11.401481	1.643344
H	-9.287963	-9.129095	1.399811
H	-7.884362	-7.182772	0.943644
H	5.870774	-6.099096	-3.123479
H	3.986416	-7.468162	-3.123479
H	6.144158	-5.388657	2.350508
H	7.023567	-4.178255	-2.350508
H	1.803355	-7.970961	-2.350508
H	3.226268	-7.508628	2.350508
H	4.394821	-9.718073	0.943644
H	5.812147	-11.654423	1.399811
H	8.283661	-11.401481	1.643344
H	9.287963	-9.129095	1.399811
H	7.884362	-7.182772	0.943644
H	-2.156655	-10.445297	-0.943644
H	-2.148172	-12.844920	-1.399811
H	0.000000	-14.093006	-1.643344
H	2.148172	-12.844920	-1.399811
H	2.156655	-10.445297	-0.943644
H	-1.164597	-8.385027	3.123479

C	8.213727	9.267128	-1.304479
C	7.656686	10.538524	-1.439512
C	6.275381	10.675418	-1.304479
C	5.463472	9.572992	-1.044077
C	6.020375	8.286336	-0.912790
N	5.197204	7.153338	-0.668035
C	5.406935	6.289530	2.157403
C	4.310864	7.085873	2.157403
C	-4.310864	-7.085873	-2.157403
C	-5.406935	-6.289530	-2.157403
N	-5.197204	-7.153338	0.668035
C	-3.826785	-7.348462	0.312046
C	-3.429828	-7.412693	-1.045322
C	-2.897080	-7.591542	1.318515
C	-1.582282	-8.012997	1.045322
C	-1.223383	-8.194358	-0.312046
C	-2.118410	-7.844548	-1.318515
C	-6.020375	-8.286336	0.912790
C	-5.463472	-9.572992	1.044077
C	-6.275381	-10.675418	1.304479
C	-7.656686	-10.538524	1.439512
C	-8.213727	-9.267128	1.304479
C	-7.416151	-8.154288	1.044077
C	5.406935	-6.289530	-2.157403
C	4.310864	-7.085873	-2.157403
C	3.429828	-7.412693	-1.045322
C	3.826785	-7.348462	0.312046
N	5.197204	-7.153338	0.668035
N	-5.197204	7.153338	-0.668035
C	-4.310864	7.085873	2.157403
C	-5.406935	6.289530	2.157403
C	-5.990015	-5.552608	-1.045322
C	-5.806261	-5.910289	0.312046
C	-6.805984	-4.438826	-1.318515
C	-7.415252	-3.695702	-0.312046
C	-7.131861	-3.980992	1.045322
C	-6.324739	-5.101202	1.318515
C	-10.792762	-2.237854	-1.044077
C	-12.092126	-2.669357	-1.304479
C	-12.388778	-4.025358	-1.439512
C	-11.351744	-4.948018	-1.304479
C	-10.046905	-4.533365	-1.044077
C	-9.741172	-3.165099	-0.912790
N	-8.409254	-2.732332	-0.668035
C	-8.071196	-1.910220	2.157403
C	-7.652534	-3.198729	2.157403
H	-1.803355	7.970961	2.350508
H	-3.226268	7.508628	-2.350508
H	7.614754	3.698714	-3.123479
H	8.334515	1.483518	-3.123479
H	7.023567	4.178255	2.350508
H	6.144158	5.388657	-2.350508
H	8.138101	-0.748070	-2.350508
H	8.138101	0.748070	2.350508
H	9.267624	5.278875	0.943644
H	11.552423	6.012332	1.399811
H	1.164597	-8.385027	3.123479
H	-8.334515	1.483518	-3.123479
H	-7.614754	3.698714	-3.123479
H	-8.138101	0.748070	2.350508
H	-8.138101	-0.748070	-2.350508
H	-6.144158	5.388657	-2.350508
H	-7.023567	4.178255	2.350508
H	-10.600511	1.176673	0.943644
H	-12.880067	1.926265	1.399811
H	-13.403245	4.354978	1.643344
H	-11.552423	6.012332	1.399811
H	-9.267624	5.278875	0.943644
H	-4.394821	9.718073	-0.943644
H	-5.812147	11.654423	-1.399811
H	-8.283661	11.401481	-1.643344
H	-9.287963	9.129095	-1.399811
H	-7.884362	7.182772	-0.943644
H	-3.986416	7.468162	3.123479
H	-5.870774	6.099096	3.123479
H	-7.023567	-4.178255	-2.350508
H	-6.144158	-5.388657	2.350508
H	-10.600511	-1.176673	-0.943644
H	-12.880067	-1.926265	-1.399811
H	-13.403245	-4.354978	-1.643344
H	-11.552423	-6.012332	-1.399811
H	-9.267624	-5.278875	-0.943644
H	-8.334515	-1.483518	3.123479
H	-7.614754	-3.698714	3.123479

[12]N-belt E = -7123.80345370, H = -7121.380942

C	1.228173	9.868754	0.314942
C	1.589231	9.696793	-1.043363
C	2.138076	9.549920	1.318081
C	3.472082	9.192284	1.043363
C	3.870748	9.160678	-0.314942
C	2.923332	9.339511	-1.318081
C	-8.248856	5.544958	-2.155711
C	-8.926502	4.371240	-2.155711
C	-9.192284	3.472082	-1.043363
C	-9.160678	3.870748	0.314942
N	-9.096444	5.251835	0.678073
C	-7.932505	5.998006	0.314942
C	-7.603054	6.224711	-1.043363
C	-7.201436	6.626588	1.318081
C	-6.224711	7.603054	1.043363
C	-5.998006	7.932505	-0.314942
C	-6.626588	7.201436	-1.318081
C	-9.549920	2.138076	-1.318081
C	-9.868754	1.228173	-0.314942
C	-9.696793	1.589231	1.043363
C	-9.339511	2.923332	1.318081
C	-10.313471	5.954485	0.912503
C	-10.330201	7.357150	1.037923
C	-11.522275	8.035186	1.286786
C	-12.729521	7.349392	1.415861
C	-12.719813	5.960990	1.286786
C	-11.536579	5.267641	1.037923
C	-12.624791	1.206379	-1.037923
C	-13.996176	1.197538	-1.286786
C	-14.698785	0.000000	-1.415861
C	-13.996176	-1.197538	-1.286786
C	-12.624791	-1.206379	-1.037923
C	-11.908970	0.000000	-0.912503
N	-10.503669	0.000000	-0.678073
C	-9.916198	-0.677646	2.155711
C	-9.916198	0.677646	2.155711
C	0.677646	9.916198	-2.155711
C	-0.677646	9.916198	-2.155711
C	-1.589231	9.696793	-1.043363
C	-8.926502	-4.371240	-2.155711
C	-8.248856	-5.544958	-2.155711
C	-7.603054	-6.224711	-1.043363
C	-7.932505	-5.998006	0.314942
N	-9.096444	-5.251835	0.678073
C	-9.160678	-3.870748	0.314942
C	-9.192284	-3.472082	-1.043363
C	-9.339511	-2.923332	1.318081
C	-9.696793	-1.589231	1.043363
C	-9.868754	-1.228173	-0.314942
C	-9.549920	-2.138076	-1.318081
C	-6.626588	-7.201436	-1.318081
C	-5.998006	-7.932505	-0.314942
C	-6.224711	-7.603054	1.043363
C	-7.201436	-6.626588	1.318081
C	-10.313471	-5.954485	0.912503
C	-10.330201	-7.357150	1.037923
C	-11.522275	-8.035186	1.286786
C	-12.729521	-7.349392	1.415861
C	-12.719813	-5.960990	1.286786
C	-11.536579	-5.267641	1.037923
C	-5.267641	-11.536579	-1.037923
C	-5.960990	-12.719813	-1.286786
C	-7.349392	-12.729521	-1.415861
C	-8.035186	-11.522275	-1.286786
C	-7.357150	-10.330201	-1.037923
C	-5.954485	-10.313471	-0.912503
N	-5.251835	-9.096444	-0.678073
C	-4.371240	-8.926502	2.155711
C	-5.544958	-8.248856	2.155711
C	8.926502	4.371240	-2.155711
C	8.248856	5.544958	-2.155711
C	7.603054	6.224711	-1.043363
C	7.932505	5.998006	0.314942
N	9.096444	5.251835	0.678073
C	9.160678	3.870748	0.314942
C	9.192284	3.472082	-1.043363
C	9.339511	2.923332	1.318081
C	9.696793	1.589231	1.043363
H	-11.571097	4.189463	0.943648
H	-12.115595	2.157367	-0.943648
H	-14.515999	2.148093	-1.378016
H	-15.767139	0.000000	-1.610716
H	-14.515999	-2.148093	-1.378016
H	-12.115595	-2.157367	-0.943648
H	-10.016492	-1.161940	3.125281
H	-10.016492	1.161940	3.125281
H	1.161940	10.016492	-3.125281
H	-1.161940	10.016492	-3.125281
H	-3.255115	9.279495	-2.350838
H	-1.820735	9.663836	2.350838
H	2.157367	12.115595	0.943648
H	2.148093	14.515999	1.378016
H	0.000000	15.767139	1.610716
H	-2.148093	14.515999	1.378016
H	-2.157367	12.115595	0.943648
H	-7.926132	9.413729	-0.943648
H	-9.118302	11.497177	-1.378016
H	-7.883569	13.654743	-1.610716
H	-5.397696	13.645270	-1.378016
H	-4.189463	11.571097	-0.943648
H	-6.014516	8.093566	3.125281
H	-4.001976	9.255507	3.125281
H	-1.161940	-10.016492	-3.125281
H	1.161940	-10.016492	-3.125281
H	-1.820735	-9.663836	2.350838
H	-3.255115	-9.279495	-2.350838
H	3.255115	-9.279495	-2.350838
H	1.820735	-9.663836	2.350838
H	-2.157367	-12.115595	0.943648
H	-2.148093	-14.515999	1.378016
H	0.000000	-15.767139	1.610716
H	2.148093	-14.515999	1.378016
H	2.157367	-12.115595	0.943648
H	7.926132	-9.413729	-0.943648
H	9.118302	-11.497177	-1.378016
H	7.883569	-13.654743	-1.610716
H	5.397696	-13.645270	-1.378016

C	-1.228173	9.868754	0.314942
N	0.000000	10.503669	0.678073
C	-2.923332	9.339511	-1.318081
C	-3.870748	9.160678	-0.314942
C	-3.472082	9.192284	1.043363
C	-2.138076	9.549920	1.318081
C	0.000000	11.908970	0.912503
C	1.206379	12.624791	1.037923
C	1.197538	13.996176	1.286786
C	0.000000	14.698785	1.415861
C	-1.197538	13.996176	1.286786
C	-1.206379	12.624791	1.037923
C	-7.357150	10.330201	-1.037923
C	-8.035186	11.522275	-1.286786
C	-7.349392	12.729521	-1.415861
C	-5.960990	12.719813	-1.286786
C	-5.267641	11.536579	-1.037923
C	-5.954485	10.313471	-0.912503
N	-5.251835	9.096444	-0.678073
C	-5.544958	8.248856	2.155711
C	-4.371240	8.926502	2.155711
C	-0.677646	-9.916198	-2.155711
C	0.677646	-9.916198	-2.155711
C	1.589231	-9.696793	-1.043363
C	1.228173	-9.868754	0.314942
N	0.000000	-10.503669	0.678073
C	-1.228173	-9.868754	0.314942
C	-1.589231	-9.696793	-1.043363
C	-2.138076	-9.549920	1.318081
C	-3.472082	-9.192284	1.043363
C	-3.870748	-9.160678	-0.314942
C	-2.923332	-9.339511	-1.318081
C	2.923332	-9.339511	-1.318081
C	3.870748	-9.160678	-0.314942
C	3.472082	-9.192284	1.043363
C	2.138076	-9.549920	1.318081
C	0.000000	-11.908970	0.912503
C	-1.206379	-12.624791	1.037923
C	-1.197538	-13.996176	1.286786
C	0.000000	-14.698785	1.415861
C	1.197538	-13.996176	1.286786
C	1.206379	-12.624791	1.037923
C	7.357150	-10.330201	-1.037923
C	8.035186	-11.522275	-1.286786
C	7.349392	-12.729521	-1.415861
C	5.960990	-12.719813	-1.286786
C	5.267641	-11.536579	-1.037923
C	5.954485	-10.313471	-0.912503
C	6.626588	-7.201436	-1.318081
C	7.603054	-6.224711	-1.043363
C	7.932505	-5.998006	0.314942
C	7.201436	-6.626588	1.318081
N	5.251835	-9.096444	-0.678073
C	5.998006	-7.932505	-0.314942
C	6.224711	-7.603054	1.043363
C	5.544958	-8.248856	2.155711
C	4.371240	-8.926502	2.155711

C	9.868754	1.228173	-0.314942
C	9.549920	2.138076	-1.318081
C	6.626588	7.201436	-1.318081
C	5.998006	7.932505	-0.314942
C	6.224711	7.603054	1.043363
C	7.201436	6.626588	1.318081
C	10.313471	5.954485	0.912503
C	11.536579	5.267641	1.037923
C	12.719813	5.960990	1.286786
C	12.729521	7.349392	1.415861
C	11.522275	8.035186	1.286786
C	10.330201	7.357150	1.037923
C	5.267641	11.536579	-1.037923
C	5.960990	12.719813	-1.286786
C	7.349392	12.729521	-1.415861
C	8.035186	11.522275	-1.286786
C	7.357150	10.330201	-1.037923
C	5.954485	10.313471	-0.912503
N	5.251835	9.096444	-0.678073
C	4.371240	8.926502	2.155711
C	5.544958	8.248856	2.155711
C	8.248856	-5.544958	-2.155711
C	8.926502	-4.371240	-2.155711
C	9.192284	-3.472082	-1.043363
C	9.160678	-3.870748	0.314942
N	9.096444	-5.251835	0.678073
C	9.549920	-2.138076	-1.318081
C	9.868754	-1.228173	-0.314942
C	9.696793	-1.589231	1.043363
C	9.339511	-2.923332	1.318081
C	10.313471	-5.954485	0.912503
C	10.330201	-7.357150	1.037923
C	11.522275	-8.035186	1.286786
C	12.729521	-7.349392	1.415861
C	12.719813	-5.960990	1.286786
C	11.536579	-5.267641	1.037923
C	12.624791	1.206379	-1.037923
C	13.996176	1.197538	-1.286786
C	14.698785	0.000000	-1.415861
C	13.996176	-1.197538	-1.286786
C	12.624791	-1.206379	-1.037923
C	11.908970	0.000000	-0.912503
N	10.503669	0.000000	-0.678073
C	9.916198	0.677646	2.155711
C	9.916198	-0.677646	2.155711
H	1.820735	9.663836	2.350838
H	3.255115	9.279495	-2.350838
H	-8.093566	6.014516	-3.125281
H	-9.255507	4.001976	-3.125281
H	-7.458760	6.408721	2.350838
H	-6.408721	7.458760	-2.350838
H	-9.663836	1.820735	-2.350838
H	-9.279495	3.255115	2.350838
H	-9.413729	7.926132	0.943648
H	-11.497177	9.118302	1.378016
H	-13.654743	7.883569	1.610716
H	-13.645270	5.397696	1.378016

H	4.189463	-11.571097	-0.943648
H	6.408721	-7.458760	-2.350838
H	7.458760	-6.408721	2.350838
H	6.014516	-8.093566	3.125281
H	4.001976	-9.255507	3.125281
H	-9.255507	-4.001976	-3.125281
H	-8.093566	-6.014516	-3.125281
H	-9.279495	-3.255115	2.350838
H	-9.663836	-1.820735	-2.350838
H	-6.408721	-7.458760	-2.350838
H	-7.458760	-6.408721	2.350838
H	-9.413729	-7.926132	0.943648
H	-11.497177	-9.118302	1.378016
H	-13.654743	-7.883569	1.610716
H	-13.645270	-5.397696	1.378016
H	-11.571097	-4.189463	0.943648
H	-4.189463	-11.571097	-0.943648
H	-5.397696	-13.645270	-1.378016
H	-7.883569	-13.654743	-1.610716
H	-9.118302	-11.497177	-1.378016
H	-7.926132	-9.413729	-0.943648
H	-4.001976	-9.255507	3.125281
H	-6.014516	-8.093566	3.125281
H	9.255507	4.001976	-3.125281
H	8.093566	6.014516	-3.125281
H	9.279495	3.255115	2.350838
H	9.663836	1.820735	-2.350838
H	6.408721	7.458760	-2.350838
H	7.458760	6.408721	2.350838
H	11.571097	4.189463	0.943648
H	13.645270	5.397696	1.378016
H	13.654743	7.883569	1.610716
H	11.497177	9.118302	1.378016
H	9.413729	7.926132	0.943648
H	4.189463	11.571097	-0.943648
H	5.397696	13.645270	-1.378016
H	7.883569	13.654743	-1.610716
H	9.118302	11.497177	-1.378016
H	7.926132	9.413729	-0.943648
H	4.001976	9.255507	3.125281
H	6.014516	8.093566	3.125281
H	8.093566	-6.014516	-3.125281
H	9.255507	-4.001976	-3.125281
H	9.663836	-1.820735	-2.350838
H	9.279495	-3.255115	2.350838
H	9.413729	-7.926132	0.943648
H	11.497177	-9.118302	1.378016
H	13.654743	-7.883569	1.610716
H	13.645270	-5.397696	1.378016
H	11.571097	-4.189463	0.943648
H	12.115595	2.157367	-0.943648
H	14.515999	2.148093	-1.378016
H	15.767139	0.000000	-1.610716
H	14.515999	-2.148093	-1.378016
H	12.115595	-2.157367	-0.943648
H	10.016492	1.161940	3.125281
H	10.016492	-1.161940	3.125281

[14]N-belt E = -8311.08457515, H = -8308.257449

C	-2.154489	1.913127	11.413072
C	-2.154694	3.235076	11.112976
C	-1.042300	4.085766	10.721358
C	0.316681	3.769742	10.963851
N	0.685222	2.706771	11.845985
C	-1.318676	5.329472	10.120323
C	-0.318507	6.235155	9.781104
C	1.040650	5.849592	9.878554
C	1.317051	4.602465	10.472354
C	0.914507	3.017559	13.219750

C	-1.279907	-14.642777	-5.721675
C	-1.400900	-14.756307	-7.106045
C	-1.272726	-13.602531	-7.878290
C	-1.032330	-12.362357	-7.289055
C	-0.914655	-12.240216	-5.890793
C	-1.320274	-11.075946	-2.939408
C	-1.044653	-11.388415	-1.593718
C	0.314077	-11.555730	-1.231435
C	1.315067	-11.259529	-2.151188
N	-0.687649	-10.971609	-5.278055

H	-2.351487	8.515007	7.722455
H	2.351788	9.438879	6.575203
H	3.124855	8.183451	8.389533
H	3.124429	6.366400	9.832809
H	2.351052	0.738291	11.454280
H	-2.351052	-0.738291	11.454280
H	-3.123734	11.020565	-4.010943
H	-3.124847	10.009421	-6.099619
H	2.353567	11.030700	-3.271918
H	-2.348310	11.365461	-1.832624

C	1.037552	1.999534	14.185432
C	1.279919	2.310970	15.522319
C	1.404886	3.633891	15.944517
C	1.278389	4.646374	14.994328
C	1.035971	4.352104	13.653560
C	-1.043140	9.854367	10.418857
C	-1.287415	10.700879	11.499021
C	-1.413081	10.200823	12.794457
C	-1.285393	8.826009	12.988288
C	-1.041106	7.965729	11.919058
C	-0.918861	8.464042	10.607307
C	-1.317862	8.699675	7.443494
C	-1.040918	9.565974	6.367745
C	0.318271	9.872739	6.114757
C	1.318231	9.205681	6.815344
N	-0.687728	7.588368	9.504557
C	-0.318057	8.164055	8.249218
C	1.041257	8.345621	7.896023
C	2.153199	7.750461	8.619828
C	2.152946	6.688978	9.462947
C	0.317067	1.367284	11.508053
C	-1.041825	0.977052	11.426841
C	1.317696	0.404378	11.423015
C	1.041825	-0.977052	11.426841
C	-0.317067	-1.367284	11.508053
C	-1.317696	-0.404378	11.423015
C	-2.152292	10.731231	-4.407452
C	-2.152934	10.140555	-5.627579
C	-1.040918	9.565974	-6.367745
C	0.318271	9.872739	-6.114757
N	0.687649	10.971609	-5.278055
C	0.319448	10.943961	-3.896799
C	-1.039467	10.953961	-3.498365
C	1.320274	11.075946	-2.939408
C	1.044653	11.388415	-1.593718
C	-0.314077	11.555730	-1.231435
C	-1.315067	11.259529	-2.151188
C	-1.317862	8.699675	-7.443494
C	-0.318057	8.164055	-8.249218
C	1.041257	8.345621	-7.896023
C	1.318231	9.205681	-6.815344
C	0.914655	12.240216	-5.890793
C	1.039646	13.410233	-5.116595
C	1.279907	14.642777	-5.721675
C	1.400900	14.756307	-7.106045
C	1.272726	13.602531	-7.878290
C	1.032330	12.362357	-7.289055
C	-1.043140	9.854367	-10.418857
C	-1.287415	10.700879	-11.499021
C	-1.413081	10.200823	-12.794457
C	-1.285393	8.826009	-12.988288
C	-1.041106	7.965729	-11.919058
C	-0.918861	8.464042	-10.607307
N	-0.687728	7.588368	-9.504557
C	2.152946	6.688978	-9.462947
C	2.153199	7.750461	-8.619828
C	-2.152934	10.140555	5.627579
C	-2.152292	10.731231	4.407452
C	-1.039467	10.953961	3.498365
C	0.319448	10.943961	3.896799
N	0.687649	10.971609	5.278055
C	-1.315067	11.259529	2.151188
C	-0.314077	11.555730	1.231435
C	1.044653	11.388415	1.593718
C	1.320274	11.075946	2.939408
C	0.914655	12.240216	5.890793
C	1.032330	12.362357	7.289055
C	1.272726	13.602531	7.878290
C	1.400900	14.756307	7.106045
C	1.279907	14.642777	5.721675
C	-0.319448	-10.943961	-3.896799
C	1.039467	-10.953961	-3.498365
C	2.152292	-10.731231	-4.407452
C	2.152934	-10.140555	-5.627579
C	-2.154694	3.235076	-11.112976
C	-2.154489	1.913127	-11.413072
C	-1.041825	0.977052	-11.426841
C	0.317067	1.367284	-11.508053
N	0.685222	2.706771	-11.845985
C	0.316681	3.769742	-10.963851
C	-1.042300	4.085766	-10.721358
C	1.317051	4.602465	-10.472354
C	1.040650	5.849592	-9.878554
C	-0.318507	6.235155	-9.781104
C	-1.318676	5.329472	-10.120323
C	-1.317696	-0.404378	-11.423015
C	-0.317067	-1.367284	-11.508053
C	1.041825	-0.977052	-11.426841
C	1.317696	0.404378	-11.423015
C	0.914507	3.017559	-13.219750
C	1.037552	1.999534	-14.185432
C	1.279919	2.310970	-15.522319
C	1.404886	3.633891	-15.944517
C	1.278389	4.646374	-14.994328
C	1.035971	4.352104	-13.653560
C	-1.035971	-4.352104	-13.653560
C	-1.278389	-4.646374	-14.994328
C	-1.404886	-3.633891	-15.944517
C	-1.279919	-2.310970	-15.522319
C	-1.037552	-1.999534	-14.185432
C	-0.914507	-3.017559	-13.219750
N	-0.685222	-2.706771	-11.845985
C	2.154694	-3.235076	-11.112976
C	2.154489	-1.913127	-11.413072
C	-2.153199	-7.750461	8.619828
C	-2.152946	-6.688978	9.462947
C	-1.040650	-5.849592	9.878554
C	0.318507	-6.235155	9.781104
N	0.687728	-7.588368	9.504557
C	0.318057	-8.164055	8.249218
C	-1.041257	-8.345621	7.896023
C	1.317862	-8.699675	7.443494
C	1.040918	-9.565974	6.367745
C	-0.318271	-9.872739	6.114757
C	-1.318231	-9.205681	6.815344
C	-1.317051	-4.602465	10.472354
C	-0.316681	-3.769742	10.963851
C	1.042300	-4.085766	10.721358
C	1.318676	-5.329472	10.120323
C	0.918861	-8.464042	10.607307
C	1.043140	-9.854367	10.418857
C	1.287415	-10.700879	11.499021
C	1.413081	-10.200823	12.794457
C	1.285393	-8.826009	12.988288
C	1.041106	-7.965729	11.919058
C	-1.037552	-1.999534	14.185432
C	-1.279919	-2.310970	15.522319
C	-1.404886	-3.633891	15.944517
C	-1.278389	-4.646374	14.994328
C	-1.035971	-4.352104	13.653560
C	-0.914507	-3.017559	13.219750
N	-0.685222	-2.706771	11.845985
C	2.154489	-1.913127	11.413072
C	2.154694	-3.235076	11.112976
C	-2.157289	-11.582594	-0.677799
C	-2.157289	-11.582594	0.677799
C	-1.044653	-11.388415	1.593718
C	0.314077	-11.555730	1.231435
N	0.681604	-12.182085	0.000000
C	-1.320274	-11.075946	2.939408
H	-2.351487	8.515007	-7.722455
H	2.351788	9.438879	-6.575203
H	0.952889	13.366386	-4.038381
H	1.371129	15.523882	-5.091267
H	1.589037	15.719410	-7.571230
H	1.358105	13.657439	-8.960776
H	0.939612	11.491547	-7.925499
H	-0.952613	10.284052	-9.429346
H	-1.377982	11.768494	-11.314410
H	-1.604348	10.865605	-13.631619
H	-1.374268	8.404488	-13.986538
H	-0.948882	6.904795	-12.113631
H	3.124429	6.366400	-9.832809
H	3.124855	8.183451	-8.389533
H	-3.124847	10.009421	6.099619
H	-3.123734	11.020565	4.010943
H	-2.348310	11.365461	1.832624
H	2.353567	11.030700	3.271918
H	0.939612	11.491547	7.925499
H	1.358105	13.657439	8.960776
H	1.589037	15.719410	7.571230
H	1.371129	15.523882	5.091267
H	0.952889	13.366386	4.038381
H	-0.940733	13.800406	-2.157880
H	-1.360070	16.200799	-2.148074
H	-1.585150	17.454209	0.000000
H	-1.360070	16.200799	2.148074
H	-0.940733	13.800406	2.157880
H	3.129068	11.669237	-1.160268
H	3.129068	11.669237	1.160268
H	-3.124429	-6.366400	-9.832809
H	-3.124855	-8.183451	-8.389533
H	2.352186	-5.643182	-10.003137
H	-2.350494	-4.315646	-10.645636
H	-2.351788	-9.438879	-6.575203
H	2.351487	-8.515007	-7.722455
H	0.948882	-6.904795	-12.113631
H	1.374268	-8.404488	-13.986538
H	1.604348	-10.865605	-13.631619
H	1.377982	-11.768494	-11.314410
H	0.952613	-10.284052	-9.429346
H	-0.952889	-13.366386	-4.038381
H	-1.371129	-15.523882	-5.091267
H	-1.589037	-15.719410	-7.571230
H	-1.358105	-13.657439	-8.960776
H	-0.939612	-11.491547	-7.925499
H	-2.353567	-11.030700	-3.271918
H	2.348310	-11.365461	-1.832624
H	3.123734	-11.020565	-4.010943
H	3.124847	-10.009421	-6.099619
H	-3.126563	3.724567	-11.090681
H	-3.126219	1.461711	-11.604381
H	2.350494	4.315646	-10.645636
H	-2.352186	5.643182	-10.003137
H	-2.351052	-0.738291	-11.454280
H	2.351052	0.738291	-11.454280
H	0.947501	0.959065	-13.900352
H	1.369512	1.498103	-16.238771
H	1.594685	3.869805	-16.987435
H	1.366752	5.688492	-15.291422
H	0.944625	5.168589	-12.948628
H	-0.944625	-5.168589	-12.948628
H	-1.366752	-5.688492	-15.291422
H	-1.594685	-3.869805	-16.987435
H	-1.369512	-1.498103	-16.238771
H	-0.947501	-0.959065	-13.900352
H	3.126563	-3.724567	-11.090681
H	3.126219	-1.461711	-11.604381
H	-3.124855	-8.183451	8.389533
H	-3.124429	-6.366400	9.832809

C	1.039646	13.410233	5.116595
C	-1.030804	14.308162	-1.206009
C	-1.271641	15.681086	-1.197200
C	-1.396548	16.384729	0.000000
C	-1.271641	15.681086	1.197200
C	-1.030804	14.308162	1.206009
C	-0.909336	13.590706	0.000000
N	-0.681604	12.182085	0.000000
C	2.157289	11.582594	-0.677799
C	2.157289	11.582594	0.677799
C	-2.152946	-6.688978	-9.462947
C	-2.153199	-7.750461	-8.619828
C	-1.041257	-8.345621	-7.896023
C	0.318057	-8.164055	-8.249218
N	0.687728	-7.588368	-9.504557
C	0.318507	-6.235155	-9.781104
C	-1.040650	-5.849592	-9.878554
C	1.318676	-5.329472	-10.120323
C	1.042300	-4.085766	-10.721358
C	-0.316681	-3.769742	-10.963851
C	-1.317051	-4.602465	-10.472354
C	-1.318231	-9.205681	-6.815344
C	-0.318271	-9.872739	-6.114757
C	1.040918	-9.565974	-6.367745
C	1.317862	-8.699675	-7.443494
C	0.918861	-8.464042	-10.607307
C	1.041106	-7.965729	-11.919058
C	1.285393	-8.826009	-12.988288
C	1.413081	-10.200823	-12.794457
C	1.287415	-10.700879	-11.499021
C	1.043140	-9.854367	-10.418857
C	-1.039646	-13.410233	-5.116595
C	-0.319448	-10.943961	3.896799
C	1.039467	-10.953961	3.498365
C	1.315067	-11.259529	2.151188
C	0.909336	-13.590706	0.000000
C	1.030804	-14.308162	-1.206009
C	1.271641	-15.681086	-1.197200
C	1.396548	-16.384729	0.000000
C	1.271641	-15.681086	1.197200
C	1.030804	-14.308162	1.206009
C	-1.032330	-12.362357	7.289055
C	-1.272726	-13.602531	7.878290
C	-1.400900	-14.756307	7.106045
C	-1.279907	-14.642777	5.721675
C	-1.039646	-13.410233	5.116595
C	-0.914655	-12.240216	5.890793
N	-0.687649	-10.971609	5.278055
C	2.152934	-10.140555	5.627579
C	2.152292	-10.731231	4.407452
H	-3.126219	1.461711	11.604381
H	-3.126563	3.724567	11.090681
H	-2.352186	5.643182	10.003137
H	2.350494	4.315646	10.645636
H	0.947501	0.959065	13.900352
H	1.369512	1.498103	16.238771
H	1.594685	3.869805	16.987435
H	1.366752	5.688492	15.291422
H	0.944625	5.168589	12.948628
H	-0.952613	10.284052	9.429346
H	-1.377982	11.768494	11.314410
H	-1.604348	10.865605	13.631619
H	-1.374268	8.404488	13.986538
H	-0.948882	6.904795	12.113631
H	2.351487	-8.515007	7.722455
H	-2.351788	-9.438879	6.575203
H	-2.350494	-4.315646	10.645636
H	2.352186	-5.643182	10.003137
H	0.952613	-10.284052	9.429346
H	1.377982	-11.768494	11.314410
H	1.604348	-10.865605	13.631619
H	1.374268	-8.404488	13.986538
H	0.948882	-6.904795	12.113631
H	-0.947501	-0.959065	13.900352
H	-1.369512	-1.498103	16.238771
H	-1.594685	-3.869805	16.987435
H	-1.366752	-5.688492	15.291422
H	-0.944625	-5.168589	12.948628
H	3.126219	-1.461711	11.604381
H	3.126563	-3.724567	11.090681
H	-3.129068	-11.669237	-1.160268
H	-3.129068	-11.669237	1.160268
H	-2.353567	-11.030700	3.271918
H	2.348310	-11.365461	1.832624
H	0.940733	-13.800406	-2.157880
H	1.360070	-16.200799	-2.148074
H	1.585150	-17.454209	0.000000
H	1.360070	-16.200799	2.148074
H	0.940733	-13.800406	2.157880
H	-0.939612	-11.491547	7.925499
H	-1.358105	-13.657439	8.960776
H	-1.589037	-15.719410	7.571230
H	-1.371129	-15.523882	5.091267
H	-0.952889	-13.366386	4.038381
H	3.124847	-10.009421	6.099619
H	3.123734	-11.020565	4.010943

[16]N-belt E = -9498.36259472, H = -9495.130978

C	4.434604	12.477539	2.152555
C	5.687214	11.958692	2.152555
C	6.468884	11.444175	1.040189
C	6.195081	11.731521	-0.319369
N	5.291732	12.775370	-0.692043
C	3.914854	12.676022	-0.319369
C	3.518062	12.666446	1.040189
C	2.950621	12.772448	-1.317646
C	1.596974	13.048574	-1.040189
C	1.234050	13.209267	0.319369
C	2.161786	12.929357	1.317646
C	7.613823	10.671050	1.317646
C	8.467757	10.212967	0.319369
C	8.097504	10.355966	-1.040189
C	6.945081	11.117889	-1.317646
C	5.831812	14.079241	-0.915188
C	4.993005	15.204648	-1.033870
C	5.526905	16.470812	-1.268483
C	6.902309	16.663647	-1.389975
C	7.738511	15.554735	-1.268483
C	7.220722	14.281897	-1.033870
C	12.136522	10.431502	1.033870
C	13.101992	11.409301	1.268483
C	12.753803	12.753803	1.389975
C	11.409301	13.101992	1.268483
C	10.431502	12.136522	1.033870
C	10.775784	10.775784	0.915188
C	10.671050	7.613823	1.317646
C	11.444175	6.468884	1.040189
C	11.731521	6.195081	-0.319369
C	11.117889	6.945081	-1.317646
N	9.777845	9.777845	0.692043
C	10.212967	8.467757	0.319369
C	10.355966	8.097504	-1.040189
C	-1.596974	-13.048574	-1.040189
C	-1.234050	-13.209267	0.319369
C	-2.161786	-12.929357	1.317646
C	-7.613823	-10.671050	1.317646
C	-8.467757	-10.212967	0.319369
C	-8.097504	-10.355966	-1.040189
C	-6.945081	-11.117889	-1.317646
C	-5.831812	-14.079241	-0.915188
C	-4.993005	-15.204648	-1.033870
C	-5.526905	-16.470812	-1.268483
C	-6.902309	-16.663647	-1.389975
C	-7.738511	-15.554735	-1.268483
C	-7.220722	-14.281897	-1.033870
C	-12.136522	-10.431502	1.033870
C	-13.101992	-11.409301	1.268483
C	-12.753803	-12.753803	1.389975
C	-11.409301	-13.101992	1.268483
C	-10.431502	-12.136522	1.033870
C	-10.775784	-10.775784	0.915188
C	-10.671050	-7.613823	1.317646
C	-11.444175	-6.468884	1.040189
C	-11.731521	-6.195081	-0.319369
C	-11.117889	-6.945081	-1.317646
N	-9.777845	-9.777845	0.692043
C	-10.212967	-8.467757	0.319369
C	-10.355966	-8.097504	-1.040189
C	-9.830694	-8.871988	-2.152555
C	-8.871988	-9.830694	-2.152555
C	5.687214	-11.958692	2.152555
C	4.434604	-12.477539	2.152555
C	3.518062	-12.666446	1.040189
C	3.914854	-12.676022	-0.319369
N	5.291732	-12.775370	-0.692043
C	6.195081	-11.731521	-0.319369
H	10.514495	7.908278	2.351625
H	11.330019	6.687760	-2.351625
H	10.226046	8.586940	-3.125719
H	8.586940	10.226046	-3.125719
H	-6.161555	11.846637	3.125719
H	-4.019960	12.733714	3.125719
H	-6.687760	11.330019	-2.351625
H	-7.908278	10.514495	2.351625
H	-1.842874	13.026868	2.351625
H	-3.282573	12.740494	-2.351625
H	-7.906906	13.449411	-0.947151
H	-8.816109	15.670620	-1.354745
H	-7.311910	17.652511	-1.573656
H	-4.846871	17.314732	-1.354745
H	-3.919143	15.101196	-0.947151
H	2.158162	15.451477	0.947151
H	2.148136	17.851544	1.354745
H	0.000000	19.106941	1.573656
H	-2.148136	17.851544	1.354745
H	-2.158162	15.451477	0.947151
H	1.159023	13.302790	-3.125719
H	-1.159023	13.302790	-3.125719
H	12.733714	-4.019960	3.125719
H	11.846637	-6.161555	3.125719
H	12.740494	-3.282573	-2.351625
H	13.026868	-1.842874	2.351625
H	10.514495	-7.908278	2.351625
H	11.330019	-6.687760	-2.351625
H	15.101196	-3.919143	-0.947151
H	17.314732	-4.846871	-1.354745
H	17.652511	-7.311910	-1.573656
H	15.670620	-8.816109	-1.354745
H	13.449411	-7.906906	-0.947151
H	12.451895	-9.399793	0.947151

C	9.830694	8.871988	-2.152555
C	8.871988	9.830694	-2.152555
C	-5.687214	11.958692	2.152555
C	-4.434604	12.477539	2.152555
C	-3.518062	12.666446	1.040189
C	-3.914854	12.676022	-0.319369
N	-5.291732	12.775370	-0.692043
C	-6.195081	11.731521	-0.319369
C	-6.468884	11.444175	1.040189
C	-6.945081	11.117889	-1.317646
C	-8.097504	10.355966	-1.040189
C	-8.467757	10.212967	0.319369
C	-7.613823	10.671050	1.317646
C	-2.161786	12.929357	1.317646
C	-1.234050	13.209267	0.319369
C	-1.596974	13.048574	-1.040189
C	-2.950621	12.772448	-1.317646
C	-5.831812	14.079241	-0.915188
C	-7.220722	14.281897	-1.033870
C	-7.738511	15.554735	-1.268483
C	-6.902309	16.663647	-1.389975
C	-5.526905	16.470812	-1.268483
C	-4.993005	15.204648	-1.033870
C	1.205632	15.958003	1.033870
C	1.196913	17.332101	1.268483
C	0.000000	18.036602	1.389975
C	-1.196913	17.332101	1.268483
C	-1.205632	15.958003	1.033870
C	0.000000	15.239260	0.915188
N	0.000000	13.827961	0.692043
C	0.677907	13.224793	-2.152555
C	-0.677907	13.224793	-2.152555
C	12.477539	-4.434604	2.152555
C	11.958692	-5.687214	2.152555
C	11.444175	-6.468884	1.040189
C	11.731521	-6.195081	-0.319369
N	12.775370	-5.291732	-0.692043
C	12.676022	-3.914854	-0.319369
C	12.666446	-3.518062	1.040189
C	12.772448	-2.950621	-1.317646
C	13.048574	-1.596974	-1.040189
C	13.209267	-1.234050	0.319369
C	12.929357	-2.161786	1.317646
C	10.671050	-7.613823	1.317646
C	10.212967	-8.467757	0.319369
C	10.355966	-8.097504	-1.040189
C	11.117889	-6.945081	-1.317646
C	14.079241	-5.831812	-0.915188
C	15.204648	-4.993005	-1.033870
C	16.470812	-5.526905	-1.268483
C	16.663647	-6.902309	-1.389975
C	15.554735	-7.738511	-1.268483
C	14.281897	-7.220722	-1.033870
C	12.136522	-10.431502	1.033870
C	13.101992	-11.409301	1.268483
C	12.753803	-12.753803	1.389975
C	11.409301	-13.101992	1.268483
C	10.431502	-12.136522	1.033870
C	10.775784	-10.775784	0.915188
N	9.777845	-9.777845	0.692043
C	8.871988	-9.830694	-2.152555
C	9.830694	-8.871988	-2.152555
C	11.958692	5.687214	2.152555
C	12.477539	4.434604	2.152555
C	12.666446	3.518062	1.040189
C	12.676022	3.914854	-0.319369
N	12.775370	5.291732	-0.692043
C	12.929357	2.161786	1.317646
C	13.209267	1.234050	0.319369
C	13.048574	1.596974	-1.040189
C	6.468884	-11.444175	1.040189
C	6.945081	-11.117889	-1.317646
C	8.097504	-10.355966	-1.040189
C	8.467757	-10.212967	0.319369
C	7.613823	-10.671050	1.317646
C	2.161786	-12.929357	1.317646
C	1.234050	-13.209267	0.319369
C	1.596974	-13.048574	-1.040189
C	2.950621	-12.772448	-1.317646
C	5.831812	-14.079241	-0.915188
C	4.993005	-15.204648	-1.033870
C	5.526905	-16.470812	-1.268483
C	6.902309	-16.663647	-1.389975
C	7.738511	-15.554735	-1.268483
C	7.220722	-14.281897	-1.033870
C	-1.205632	-15.958003	1.033870
C	-1.196913	-17.332101	1.268483
C	0.000000	-18.036602	1.389975
C	1.196913	-17.332101	1.268483
C	1.205632	-15.958003	1.033870
C	0.000000	-15.239260	0.915188
N	0.000000	-13.827961	0.692043
C	-0.677907	-13.224793	-2.152555
C	0.677907	-13.224793	-2.152555
C	-12.477539	4.434604	2.152555
C	-11.958692	5.687214	2.152555
C	-11.444175	6.468884	1.040189
C	-11.731521	6.195081	-0.319369
N	-12.775370	5.291732	-0.692043
C	-12.676022	3.914854	-0.319369
C	-12.666446	3.518062	1.040189
C	-12.772448	2.950621	-1.317646
C	-13.048574	1.596974	-1.040189
C	-13.209267	1.234050	0.319369
C	-12.929357	2.161786	1.317646
C	-10.671050	7.613823	1.317646
C	-10.212967	8.467757	0.319369
C	-10.355966	8.097504	-1.040189
C	-11.117889	6.945081	-1.317646
C	-14.079241	5.831812	-0.915188
C	-15.204648	4.993005	-1.033870
C	-16.470812	5.526905	-1.268483
C	-16.663647	6.902309	-1.389975
C	-15.554735	7.738511	-1.268483
C	-14.281897	7.220722	-1.033870
C	-10.431502	12.136522	1.033870
C	-11.409301	13.101992	1.268483
C	-12.753803	12.753803	1.389975
C	-13.101992	11.409301	1.268483
C	-12.136522	10.431502	1.033870
C	-10.775784	10.775784	0.915188
N	-9.777845	9.777845	0.692043
C	-8.871988	9.830694	-2.152555
C	-9.830694	8.871988	-2.152555
C	-11.958692	-5.687214	2.152555
C	-12.477539	-4.434604	2.152555
C	-12.666446	-3.518062	1.040189
C	-12.676022	-3.914854	-0.319369
N	-12.775370	-5.291732	-0.692043
C	-12.929357	-2.161786	1.317646
C	-13.209267	-1.234050	0.319369
C	-13.048574	-1.596974	-1.040189
C	-12.772448	-2.950621	-1.317646
C	-14.079241	-5.831812	-0.915188
C	-14.281897	-7.220722	-1.033870
C	-15.554735	-7.738511	-1.268483
C	-16.663647	-6.902309	-1.389975
C	-16.470812	-5.526905	-1.268483
C	-15.204648	-4.993005	-1.033870
C	-15.958003	1.205632	1.033870
H	14.141909	-11.103986	1.354745
H	13.510647	-13.510647	1.573656
H	11.103986	-14.141909	1.354745
H	9.399793	-12.451895	0.947151
H	8.586940	-10.226046	-3.125719
H	10.226046	-8.586940	-3.125719
H	11.846637	6.161555	3.125719
H	12.733714	4.019960	3.125719
H	13.026868	1.842874	2.351625
H	12.740494	3.282573	-2.351625
H	13.449411	7.906906	-0.947151
H	15.670620	8.816109	-1.354745
H	17.652511	7.311910	-1.573656
H	17.314732	4.846871	-1.354745
H	15.101196	3.919143	-0.947151
H	15.451477	-2.158162	0.947151
H	17.851544	-2.148136	1.354745
H	19.106941	0.000000	1.573656
H	17.851544	2.148136	1.354745
H	15.451477	2.158162	0.947151
H	13.302790	-1.159023	-3.125719
H	13.302790	1.159023	-3.125719
H	-4.019960	-12.733714	3.125719
H	-6.161555	-11.846637	3.125719
H	-3.282573	-12.740494	-2.351625
H	-1.842874	-13.026868	2.351625
H	-7.908278	-10.514495	2.351625
H	-6.687760	-11.330019	-2.351625
H	-3.919143	-15.101196	-0.947151
H	-4.846871	-17.314732	-1.354745
H	-7.311910	-17.652511	-1.573656
H	-8.816109	-15.670620	-1.354745
H	-7.906906	-13.449411	-0.947151
H	-12.451895	-9.399793	0.947151
H	-14.141909	-11.103986	1.354745
H	-13.510647	-13.510647	1.573656
H	-11.103986	-14.141909	1.354745
H	-9.399793	-12.451895	0.947151
H	-10.514495	-7.908278	2.351625
H	-11.330019	-6.687760	-2.351625
H	-10.226046	-8.586940	-3.125719
H	-8.586940	-10.226046	-3.125719
H	6.161555	-11.846637	3.125719
H	4.019960	-12.733714	3.125719
H	6.687760	-11.330019	-2.351625
H	7.908278	-10.514495	2.351625
H	1.842874	-13.026868	2.351625
H	3.282573	-12.740494	-2.351625
H	3.919143	-15.101196	-0.947151
H	4.846871	-17.314732	-1.354745
H	7.311910	-17.652511	-1.573656
H	8.816109	-15.670620	-1.354745
H	7.906906	-13.449411	-0.947151
H	-2.158162	-15.451477	0.947151
H	-2.148136	-17.851544	1.354745
H	0.000000	-19.106941	1.573656
H	2.148136	-17.851544	1.354745
H	2.158162	-15.451477	0.947151
H	-1.159023	-13.302790	-3.125719
H	1.159023	-13.302790	-3.125719
H	-12.733714	4.019960	3.125719
H	-11.846637	6.161555	3.125719
H	-12.740494	3.282573	-2.351625
H	-13.026868	1.842874	2.351625
H	-10.514495	7.908278	2.351625
H	-11.330019	6.687760	-2.351625
H	-15.101196	3.919143	-0.947151
H	-17.314732	4.846871	-1.354745
H	-17.652511	7.311910	-1.573656
H	-15.670620	8.816109	-1.354745

C	12.772448	2.950621	-1.317646	C	-17.332101	1.196913	1.268483	H	-13.449411	7.906906	-0.947151
C	14.079241	5.831812	-0.915188	C	-18.036602	0.000000	1.389975	H	-9.399793	12.451895	0.947151
C	14.281897	7.220722	-1.033870	C	-17.332101	-1.196913	1.268483	H	-11.103986	14.141909	1.354745
C	15.554735	7.738511	-1.268483	C	-15.958003	-1.205632	1.033870	H	-13.510647	13.510647	1.573656
C	16.663647	6.902309	-1.389975	C	-15.239260	0.000000	0.915188	H	-14.141909	11.103986	1.354745
C	16.470812	5.526905	-1.268483	N	-13.827961	0.000000	0.692043	H	-12.451895	9.399793	0.947151
C	15.204648	4.993005	-1.033870	C	-13.224793	0.677907	-2.152555	H	-8.586940	10.226046	-3.125719
C	15.958003	-1.205632	1.033870	C	-13.224793	-0.677907	-2.152555	H	-10.226046	8.586940	-3.125719
C	17.332101	-1.196913	1.268483	H	4.019960	12.733714	3.125719	H	-11.846637	-6.161555	3.125719
C	18.036602	0.000000	1.389975	H	6.161555	11.846637	3.125719	H	-12.733714	-4.019960	3.125719
C	17.332101	1.196913	1.268483	H	3.282573	12.740494	-2.351625	H	-13.026868	-1.842874	2.351625
C	15.958003	1.205632	1.033870	H	1.842874	13.026868	2.351625	H	-12.740494	-3.282573	-2.351625
C	15.239260	0.000000	0.915188	H	7.908278	10.514495	2.351625	H	-13.449411	-7.906906	-0.947151
N	13.827961	0.000000	0.692043	H	6.687760	11.330019	-2.351625	H	-15.670620	-8.816109	-1.354745
C	13.224793	-0.677907	-2.152555	H	3.919143	15.101196	-0.947151	H	-17.652511	-7.311910	-1.573656
C	13.224793	0.677907	-2.152555	H	4.846871	17.314732	-1.354745	H	-17.314732	-4.846871	-1.354745
C	-4.434604	-12.477539	2.152555	H	7.311910	17.652511	-1.573656	H	-15.101196	-3.919143	-0.947151
C	-5.687214	-11.958692	2.152555	H	8.816109	15.670620	-1.354745	H	-15.451477	2.158162	0.947151
C	-6.468884	-11.444175	1.040189	H	7.906906	13.449411	-0.947151	H	-17.851544	2.148136	1.354745
C	-6.195081	-11.731521	-0.319369	H	12.451895	9.399793	0.947151	H	-19.106941	0.000000	1.573656
N	-5.291732	-12.775370	-0.692043	H	14.141909	11.103986	1.354745	H	-17.851544	-2.148136	1.354745
C	-3.914854	-12.676022	-0.319369	H	13.510647	13.510647	1.573656	H	-15.451477	-2.158162	0.947151
C	-3.518062	-12.666446	1.040189	H	11.103986	14.141909	1.354745	H	-13.302790	1.159023	-3.125719
C	-2.950621	-12.772448	-1.317646	H	9.399793	12.451895	0.947151	H	-13.302790	-1.159023	-3.125719

3 E = -825.902052305, H = -825.594096

C	-3.665883	-1.930679	-0.308450	C	0.000603	1.547039	-0.138323	C	3.286049	-1.258617	-1.473415
C	-2.814441	-1.947400	0.790602	C	1.210714	-0.590114	-0.409325	H	1.758959	-0.043777	-2.404823
C	-3.287119	-1.256207	-1.473272	C	1.563973	-1.298499	0.760722	C	3.664214	-1.933663	-0.308727
C	-1.565125	-1.297083	0.760905	H	1.171153	-1.613858	2.863640	H	3.101540	-2.486384	1.691044
H	-3.103687	-2.483522	1.691425	C	1.209130	2.253482	0.015533	C	0.001921	4.316477	0.446519
C	-2.063360	-0.591393	-1.518505	C	-1.207226	2.254770	0.014662	H	2.150345	4.134049	0.415961
C	-1.211288	-0.589154	-0.409238	C	2.062843	-0.592792	-1.518552	H	-2.146667	4.136369	0.414452
C	-0.676627	-1.406741	1.916509	C	2.812744	-1.949875	0.790306	H	3.944953	-1.242894	-2.337095
H	-1.758996	-0.042817	-2.404883	C	1.200510	3.616978	0.302027	H	4.619787	-2.448620	-0.261196
N	0.000022	0.170176	-0.453881	H	2.155866	1.735769	-0.085294	H	-3.945977	-1.240129	-2.336980
C	0.675490	-1.407406	1.916408	C	-1.197299	3.618291	0.301200	H	-4.621888	-2.444824	-0.260834
H	-1.172357	-1.612703	2.863811	H	-2.154482	1.738139	-0.086844	H	0.002355	5.379485	0.669273

4 E = -1419.55538083, H = -1419.045518

N	-2.799591	-0.041196	0.404815	H	-6.495507	2.662483	0.760647	C	2.638187	2.378217	-0.790592
C	-3.411941	1.220712	0.581976	C	-5.075366	-2.982210	0.619222	C	4.250076	-1.919442	-0.981957
C	-1.390315	-0.142594	0.187609	H	-4.135757	-1.632258	2.022798	C	4.540969	-2.589504	1.701374
C	-2.637777	2.378395	0.789134	C	-4.541529	-2.589051	-1.701558	C	5.410736	2.593766	-0.772325
C	-4.813894	1.351057	0.573325	C	-2.936750	-0.844092	-2.410840	H	5.437829	0.480908	-0.402890
C	-3.572821	-1.177849	0.008693	C	1.390126	-0.142606	-0.187613	C	3.251425	3.613600	-0.988913
C	-0.526998	-0.162632	1.279743	C	1.717873	-0.264779	2.313057	H	1.556336	2.313908	-0.796207
C	-0.870641	-0.182197	-1.127772	H	0.958240	-0.153151	-2.276781	C	5.075449	-2.982014	-0.619366
C	-3.250662	3.613909	0.987594	H	-1.262130	0.091193	-3.235238	H	4.136213	-1.631696	-2.022833
H	-1.555924	2.313965	0.793481	C	-5.226299	-3.311861	-0.731156	C	5.225975	-3.312069	0.730964
C	-5.410341	2.594224	0.774015	H	-4.648975	-2.854381	-2.750618	H	4.648183	-2.855073	2.750398
H	-5.438085	0.481178	0.405752	H	-3.373414	-0.911035	-3.405930	C	4.641022	3.737943	-0.984512
C	-4.249916	-1.919707	0.981872	N	2.799426	-0.041246	-0.404818	H	6.495888	2.661968	-0.757716
C	-3.687973	-1.522549	-1.356603	C	2.936367	-0.844423	2.410769	H	2.626203	4.489143	-1.146658
C	0.870443	-0.181961	1.127773	H	1.262058	0.091362	3.235248	H	5.605727	-3.542901	-1.383876
H	-0.958428	-0.152592	2.276783	C	3.411979	1.220572	-0.581921	H	5.873797	-4.133584	1.024055
C	0.526814	-0.162963	-1.279736	C	3.572612	-1.177926	-0.008770	H	-5.874204	-4.133293	-1.024296
C	-1.718064	-0.264858	-2.313069	C	3.687513	-1.522894	1.356485	H	-5.605395	-3.543335	1.383729
C	-4.640275	3.738403	0.984740	H	3.373015	-0.911574	3.405855	H	-5.111692	4.704271	1.140918
H	-2.625202	4.489493	1.144161	C	4.813946	1.350781	-0.571667	H	5.112697	4.703696	-1.140624

N-Belt **2** (B3LYP/6-31G(d)) E = -4249.03532993

N	-0.745899	-5.476122	0.630555	C	5.321413	-5.645223	-0.802471	C	-6.416296	6.489844	0.929204
C	-0.935285	-6.879470	0.720215	C	6.796299	-3.744783	-0.769720	H	-4.326241	6.074135	0.766341
C	-1.841070	-4.615992	0.297742	C	4.684022	-1.066472	-1.318718	C	-7.898595	4.594817	0.898356
C	-2.228200	-7.431091	0.802471	C	4.794966	-0.225023	1.318723	H	-6.967386	2.675911	0.707539
C	0.155072	-7.758159	0.769720	C	-8.828518	-2.311753	-0.929204	C	-0.913679	4.759778	1.054811

C	0.538994	-4.940132	0.297733
C	-2.592359	-4.040051	1.318723
C	-2.155535	-4.343909	-1.054798
C	-2.412222	-8.801598	0.929204
H	-3.097235	-6.783702	0.766341
C	-0.029930	-9.137792	0.898356
H	1.166286	-7.371889	0.707539
C	1.418419	-4.589718	1.318718
C	0.913679	-4.759778	-1.054811
C	-3.665249	-3.171158	1.054811
H	-2.313075	-4.255749	2.346194
C	-3.265602	-3.523246	-1.318718
C	-1.340832	-4.825622	-2.167725
C	-1.317334	-9.672736	0.979409
H	-3.412425	-9.219926	0.990363
H	0.845127	-9.777380	0.930726
C	2.684168	-4.038703	1.054798
H	1.092621	-4.725286	2.346188
C	2.202607	-4.265074	-1.318723
C	0.000000	-5.006657	-2.167748
C	-4.008783	-2.936848	-0.297733
C	-4.335892	-2.503328	2.167748
H	-3.545908	-3.308881	-2.346188
H	-1.860402	-4.933197	-3.118181
O	-1.612563	-11.006771	1.106042
C	3.077031	-3.902409	-0.297742
C	3.508696	-3.574005	2.167725
H	2.529050	-4.131056	-2.346194
H	0.472324	-5.248187	-3.118262
N	-5.115410	-2.092094	-0.630555
C	-4.849527	-1.251617	2.167725
H	-4.308901	-3.033138	3.118262
C	-0.532008	-11.919241	1.157484
N	4.369512	-3.384028	-0.630555
C	4.335892	-2.503328	2.167748
H	3.342073	-4.077754	3.118181
C	-6.425438	-2.629755	-0.720215
C	-4.918101	-0.713583	-0.297742
C	-4.839703	-0.305206	1.054798
H	-5.202475	-0.855443	3.118181
H	0.072439	-11.886684	0.240163
H	0.121109	-11.728688	2.020649
H	-0.978057	-12.910960	1.257691
C	5.490153	-4.249715	-0.720215
C	4.547776	-2.003284	-0.297733
C	4.578928	-1.588620	1.054811
H	4.781225	-2.215049	3.118262
C	-7.549613	-1.785867	-0.802471
C	-6.641227	-4.013376	-0.769720
C	-4.794966	0.225023	-1.318723
C	-4.684022	1.066472	1.318718
H	-7.423476	-0.709567	-0.766341
C	-7.928525	-4.542976	-0.898356
H	-5.801100	-4.695978	-0.707539
C	-4.578928	1.588620	-1.054811
H	-4.842125	-0.124693	-2.346194
C	-4.547776	2.003284	0.297733
H	-4.638529	1.416406	2.346188
C	6.416296	-6.489844	-0.929204
H	4.326241	-6.074135	-0.766341
C	7.898595	-4.594817	-0.898356
H	6.967386	-2.675911	-0.707539
C	4.839703	0.305206	-1.054798
H	4.638529	-1.416406	-2.346188
C	4.918101	0.713583	0.297742
H	4.842125	0.124693	2.346194
C	-9.035503	-3.695523	-0.979409
H	-9.690903	-1.654716	-0.990363
H	-8.044896	-5.620591	-0.930726
C	-4.335892	2.503328	-2.167748
N	-4.369512	3.384028	0.630555
C	7.718168	-5.977213	-0.979409
H	6.278478	-7.565210	-0.990363
H	8.890023	-4.156789	-0.930726
C	4.849527	1.251617	-2.167725
N	5.115410	2.092094	0.630555
O	-10.338425	-4.106865	-1.106042
C	-3.508696	3.574005	-2.167725
H	-4.781225	2.215049	-3.118262
C	-5.490153	4.249715	0.720215
C	-3.077031	3.902409	0.297742
O	8.725862	-6.899906	-1.106042
C	4.335892	2.503328	-2.167748
H	5.202475	0.855443	-3.118181
C	6.425438	2.629755	0.720215
C	4.008783	2.936848	0.297733
C	-10.588369	-5.498888	-1.157484
C	-2.684168	4.038703	-1.054798
H	-3.342073	4.077754	-3.118181
C	-5.321413	5.645223	0.802471
C	-6.796299	3.744783	0.769720
C	-2.202607	4.265074	1.318723
C	10.056361	-6.420353	-1.157484
C	3.665249	3.171158	-1.054811
H	4.308901	3.033138	-3.118262
C	7.549613	1.785867	0.802471
C	6.641227	4.013376	0.769720
C	3.265602	3.523246	1.318718
H	-10.257951	-6.006076	-0.240163
H	-10.096787	-5.969228	-2.020649
H	-11.670247	-5.608458	-1.257691
C	-1.418419	4.589718	-1.318718
H	-2.529050	4.131056	2.346194
H	10.330390	-5.880608	-0.240163
H	10.217896	-5.759460	-2.020649
H	10.692191	-7.302502	-1.257691
C	2.592359	4.040051	-1.318723
C	8.828518	2.311753	0.929204
H	7.423476	0.709567	0.766341
C	7.928525	4.542976	0.898356
H	5.801100	4.695978	0.707539
C	2.155535	4.343909	1.054798
H	3.545908	3.308881	2.346188
C	-0.538994	4.940132	-0.297733
H	-1.092621	4.725286	-2.346188
C	-7.718168	5.977213	0.979409
H	-6.278478	7.565210	0.990363
H	-8.890023	4.156789	0.930726
C	0.000000	5.006657	2.167748
C	1.841070	4.615992	-0.297742
H	2.313075	4.255749	-2.346194
C	9.035503	3.695523	0.979409
H	9.690903	1.654716	0.990363
H	8.044896	5.620591	0.930726
C	1.340832	4.825622	2.167725
N	0.745899	5.476122	-0.630555
O	-8.725862	6.899906	1.106042
H	-0.472324	5.248187	3.118262
O	10.338425	4.106865	1.106042
H	1.860402	4.933197	3.118181
C	0.935285	6.879470	-0.720215
C	-10.056361	6.420353	1.157484
C	10.588369	5.498888	1.157484
C	2.228200	7.431091	-0.802471
C	-0.155072	7.758159	-0.769720
H	-10.330390	5.880608	0.240163
H	-10.217896	5.759460	2.020649
H	-10.692191	7.302502	1.257691
H	10.257951	6.006076	0.240163
H	10.096787	5.969228	2.020649
H	11.670247	5.608458	1.257691
C	2.412222	8.801598	-0.929204
H	3.097235	6.783702	-0.766341
C	0.029930	9.137792	-0.898356
H	-1.166286	7.371889	-0.707539
C	1.317334	9.672736	-0.979409
H	3.412425	9.219926	-0.990363
H	-0.845127	9.777380	-0.930726
O	1.612563	11.006771	-1.106042
C	0.532008	11.919241	-1.157484
H	-0.072439	11.886684	-0.240163
H	-0.121109	11.728688	-2.020649
H	0.978057	12.910960	-1.257691

N-Belt **2**$^+$ (UB3LYP/6-31G(d)) E = -4248.83612625

N	1.336745	5.361544	0.614066
C	1.640455	6.580205	1.256165
C	2.333567	4.389167	0.301459
C	2.974013	6.927315	1.564896
C	0.634612	7.512377	1.565674
C	0.000000	4.971625	0.301316
C	3.017189	3.727208	1.320276
C	2.610797	4.084586	-1.051888
C	3.272915	8.134987	2.174208
H	3.784227	6.253185	1.314738
C	0.935942	8.727964	2.178884
H	-0.398320	7.300950	1.316614
C	-0.914373	4.708132	1.320193
C	-0.387572	4.832468	-1.051941
C	3.991254	2.751881	1.051941
H	2.772885	3.964185	2.351784
C	-4.512224	6.039228	-1.564896
C	-6.188603	4.305779	-1.565674
C	-4.534548	1.562195	-1.320193
C	-4.736451	0.749358	1.320276
C	8.681563	1.233066	-2.174208
H	7.307530	-0.150645	-1.314738
C	8.026609	3.553432	-2.178884
H	6.123648	3.995430	-1.316614
C	4.378826	-2.080586	-1.051941
H	4.819527	-0.419296	-2.351784
C	4.305553	-2.485812	0.301316
H	4.453442	-1.892929	2.351630
C	-5.408648	6.901921	-2.174208
H	-3.523303	6.403829	-1.314738
C	-7.090667	5.174532	-2.178884
H	-6.521968	3.305520	-1.316614
C	5.408648	-6.901921	2.174208
H	3.523303	-6.403829	1.314738
C	7.090667	-5.174532	2.178884
H	6.521968	-3.305520	1.316614
C	0.387572	-4.832468	1.051941
H	2.046642	-4.383481	2.351784
H	-9.411945	6.770677	-2.538308
H	-8.855297	6.208905	-4.145129
H	-9.280563	7.924880	-3.894341
C	-3.017189	-3.727208	-1.320276
C	-8.681563	-1.233066	2.174208
H	-7.307530	0.150645	1.314738
C	-8.026609	-3.553432	2.178884
H	-6.123648	-3.995430	1.316614
C	-2.610797	-4.084586	1.051888
H	-3.866046	-2.910329	2.351630

C	3.620175	3.145937	-1.320193
C	1.859314	4.663136	-2.163837
C	2.259964	9.051122	2.497223
H	4.300602	8.395989	2.406336
H	0.126235	9.415331	2.395343
C	-2.231957	4.303309	1.051888
H	-0.587396	4.803259	2.351630
C	-1.719262	4.476566	-1.320276
C	0.547584	4.990383	-2.163903
C	4.305553	2.485812	-0.301316
C	4.595590	2.020969	2.163903
H	3.866046	2.910329	-2.351630
H	2.391980	4.724077	-3.110881
O	2.665275	10.203070	3.098422
C	-2.634347	4.215512	-0.301459
C	-3.108737	3.941781	2.163837
H	-2.046642	4.383481	-2.351784
H	0.105910	5.294554	-3.110862
N	5.311606	1.523117	-0.614066
C	4.968051	0.721355	2.163837
H	4.638173	2.555557	3.110862
C	1.681902	11.172938	3.431676
N	-3.974861	3.838427	-0.614066
C	-4.048006	2.969413	2.163903
H	-2.895181	4.433554	3.110881
C	6.518852	1.869426	-1.256165
C	4.967913	0.173655	-0.301459
C	4.842754	-0.218723	1.051888
H	5.287161	0.290523	3.110881
H	1.157606	11.536322	2.538308
H	0.949421	10.773364	4.145129
H	2.222866	11.999643	3.894341
C	-4.878397	4.710778	-1.256165
C	-4.305553	2.485812	-0.301316
C	-4.378826	2.080586	1.051941
H	-4.532264	2.738997	3.110862
C	7.486237	0.888087	-1.564896
C	6.823215	3.206598	-1.565674
C	4.736451	-0.749358	-1.320276
C	4.534548	-1.562195	1.320193
C	-4.842754	0.218723	-1.051888
H	-4.453442	1.892929	-2.351630
C	-4.967913	-0.173655	0.301459
H	-4.819527	0.419296	2.351784
C	8.968484	2.568375	-2.497223
H	9.421441	0.473564	-2.406336
H	8.217033	4.598343	-2.395343
C	4.048006	-2.969413	-2.163903
N	3.974861	-3.838427	0.614066
C	-6.708520	6.482747	-2.497223
H	-5.120839	7.922425	-2.406336
H	-8.090798	4.816988	-2.395343
C	-4.968051	-0.721355	-2.163837
N	-5.311606	-1.523117	0.614066
O	10.168755	2.793339	-3.098422
C	3.108737	-3.941781	-2.163837
H	4.532264	-2.738997	-3.110862
C	4.878397	-4.710778	1.256165
C	2.634347	-4.215512	0.301459
O	-7.503480	7.409731	-3.098422
C	-4.595590	-2.020969	-2.163903
H	-5.287161	-0.290523	-3.110881
C	-6.518852	-1.869426	1.256165
C	-4.305553	-2.485812	0.301316
C	10.516999	4.129899	-3.431676
C	2.231957	-4.303309	-1.051888
H	2.895181	-4.433554	-3.110881
C	4.512224	-6.039228	1.564896
C	6.188603	-4.305779	1.565674
C	1.719262	-4.476566	1.320276
C	-8.835097	7.043038	-3.431676
C	-3.991254	-2.751881	-1.051941
H	-4.638173	-2.555557	-3.110862
C	-7.486237	-0.888087	1.564896
C	-6.823215	-3.206598	1.565674
C	-3.620175	-3.145937	1.320193
H	10.569551	4.765645	-2.538308
H	9.804718	4.564460	-4.145129
H	11.503429	4.074763	-3.894341
C	0.914373	-4.708132	-1.320193
C	0.000000	-4.971625	-0.301316
H	0.587396	-4.803259	-2.351630
C	6.708520	-6.482747	2.497223
H	5.120839	-7.922425	2.406336
H	8.090798	-4.816988	2.395343
C	-0.547584	-4.990383	2.163903
C	-2.333567	-4.389167	-0.301459
H	-2.772885	-3.964185	-2.351784
C	-8.968484	-2.568375	2.497223
H	-9.421441	-0.473564	2.406336
H	-8.217033	-4.598343	2.395343
C	-1.859314	-4.663136	2.163837
N	-1.336745	-5.361544	-0.614066
O	7.503480	-7.409731	3.098422
H	-0.105910	-5.294554	3.110862
O	-10.168755	-2.793339	3.098422
H	-2.391980	-4.724077	3.110881
C	-1.640455	-6.580205	-1.256165
C	8.835097	-7.043038	3.431676
C	-10.516999	-4.129899	3.431676
C	-2.974013	-6.927315	-1.564896
C	-0.634612	-7.512377	-1.565674
H	9.411945	-6.770677	2.538308
H	8.855297	-6.208905	4.145129
H	9.280563	-7.924880	3.894341
H	-10.569551	-4.765645	2.538308
H	-9.804718	-4.564460	4.145129
H	-11.503429	-4.074763	3.894341
C	-3.272915	-8.134987	-2.174208
H	-3.784227	-6.253185	-1.314738
C	-0.935942	-8.727964	-2.178884
H	0.398320	-7.300950	-1.316614
C	-2.259964	-9.051122	-2.497223
H	-4.300602	-8.395989	-2.406336
H	-0.126235	-9.415331	-2.395343
O	-2.665275	-10.203070	-3.098422
C	-1.681902	-11.172938	-3.431676
H	-1.157606	-11.536322	-2.538308
H	-0.949421	-10.773364	-4.145129
H	-2.222866	-11.999643	-3.894341

N-Belt $\mathbf{2}^{2+}$ (UB3LYP/6-31G(d)_T) E = -4248.57604166

N	0.241275	5.523506	0.589627
C	0.295860	6.760075	1.248364
C	1.413276	4.766839	0.275741
C	1.537952	7.360832	1.569743
C	-0.881488	7.473288	1.560411
C	-0.992497	4.872357	0.276437
C	2.212197	4.259542	1.299322
C	1.741133	4.520116	-1.077841
C	1.588687	8.590200	2.196156
H	2.465532	6.865401	1.311775
C	-0.830420	8.710494	2.190530
H	-1.849264	7.066404	1.294656
C	-1.832130	4.437310	1.300759
C	-1.341055	4.654044	-1.076800
C	3.356131	3.489904	1.034604
H	1.927170	4.448193	2.330211
C	2.921409	3.806327	-1.341652
C	0.891324	4.934754	-2.191797
C	0.407391	9.283827	2.526107
H	2.541042	9.050636	2.437623
H	-1.758617	9.225978	2.407103
C	-3.038367	3.769286	1.036994
H	-1.531190	4.601385	2.331326
C	-2.578752	4.044737	-1.339639
C	-0.459273	4.993290	-2.191422
C	3.718632	3.292869	-0.319103
C	4.094450	2.901410	2.150422
C	-5.626347	5.024114	-1.548008
C	-6.912955	2.977436	-1.589411
C	-4.750663	0.622626	-1.319926
C	-4.783692	-0.206160	1.323845
C	8.246166	2.928981	-2.202494
H	7.167871	1.293978	-1.368997
C	7.160011	5.089044	-2.156189
H	5.219372	5.144986	-1.272743
C	4.701257	-1.169147	-1.051273
H	4.796724	0.542096	-2.356590
C	4.711951	-1.575131	0.303017
H	4.736131	-0.958728	2.352597
C	-6.673900	5.691286	-2.155091
H	-4.736660	5.580648	-1.279875
C	-7.968653	3.649386	-2.197870
H	-7.034183	1.926682	-1.356215
C	-4.786315	-0.754669	-1.047412
H	-4.736132	0.958729	-2.352597
C	-4.829638	-1.158361	0.307020
H	-4.796728	-0.542097	2.356589
C	8.271227	4.304174	-2.499784
H	9.116103	2.330967	-2.453654
H	7.150688	6.155165	-2.350292
C	4.553287	-2.109346	-2.160147
N	4.657154	-2.968039	0.623465
C	-7.860267	5.015869	-2.496965
H	-6.604103	6.752541	-2.370435
C	6.673898	-5.691285	2.155092
H	4.736658	-5.580648	1.279876
C	7.968650	-3.649385	2.197871
H	7.034179	-1.926681	1.356215
C	1.341052	-4.654043	1.076801
H	2.885516	-3.891156	2.370086
H	-10.570364	4.758298	-2.561125
H	-9.900785	4.344498	-4.172779
H	-10.655040	5.939777	-3.897672
C	-2.212198	-4.259542	-1.299323
C	-8.246169	-2.928976	2.202491
H	-7.167869	-1.293975	1.368996
C	-7.160020	-5.089042	2.156185
H	-5.219381	-5.144988	1.272740
C	-1.741136	-4.520119	1.077840
H	-3.213341	-3.627011	2.372241
C	0.992494	-4.872357	-0.276437
H	1.531188	-4.601387	-2.331326
C	7.860264	-5.015868	2.496966
H	6.604102	-6.752540	2.370437
H	8.873642	-3.099659	2.428419
C	0.459269	-4.993290	2.191422
C	-1.413279	-4.766841	-0.275741
H	-1.927170	-4.448192	-2.330211
C	-8.271233	-4.304169	2.499780
H	-9.116104	-2.330959	2.453651
H	-7.150700	-6.155163	2.350287

H	3.213337	3.627009	-2.372242	H	-8.873646	3.099661	-2.428417	C	-0.891328	-4.934756	2.191796
H	1.401722	5.100733	-3.138090	C	-4.721584	-1.704080	-2.156358	N	-0.241277	-5.523508	-0.589626
O	0.574776	10.472512	3.139216	N	-4.897309	-2.550844	0.627401	O	8.816989	-5.764864	3.088869
C	-3.417297	3.603501	-0.316440	O	9.395460	4.759680	-3.095852	H	0.954074	-5.202917	3.137331
C	-3.824475	3.247595	2.153428	C	3.824472	-3.247597	-2.153427	O	-9.395468	-4.759673	3.095847
H	-2.885520	3.891157	-2.370085	H	4.983831	-1.793809	-3.108175	H	-1.401725	-5.100734	3.138089
H	-0.954077	5.202917	-3.137331	C	5.714656	-3.641715	1.261500	C	-0.295857	-6.760077	-1.248362
N	4.897307	2.550843	-0.627403	C	3.417294	-3.603501	0.316441	C	10.053961	-5.150828	3.445366
C	4.721585	1.704080	2.156356	O	-8.816991	5.764866	-3.088866	C	-9.496913	-6.146431	3.412891
H	4.030220	3.439663	3.093750	C	-4.094450	-2.901410	-2.150423	C	-1.537946	-7.360844	-1.569738
C	-0.574501	11.244400	3.490014	H	-5.123532	-1.352188	-3.104148	C	0.881495	-7.473283	-1.560411
N	-4.657157	2.968038	-0.623465	C	-6.010570	-3.128812	1.264463	H	10.570362	-4.758295	2.561127
C	-4.553290	2.109345	2.160147	C	-3.718634	-3.292870	0.319102	H	9.900781	-4.344495	4.172781
H	-3.712995	3.778263	3.096661	C	9.496901	6.146439	-3.412897	H	10.655038	-5.939773	3.897675
C	6.010566	3.128812	-1.264465	C	3.038365	-3.769287	-1.036994	H	-9.441141	-6.764374	2.508613
C	4.829637	1.158361	-0.307021	H	3.712992	-3.778265	-3.096661	H	-8.713226	-6.450084	4.117479
C	4.786315	0.754669	1.047411	C	5.626344	-5.024114	1.548009	H	-10.474209	-6.271411	3.879350
H	5.123532	1.352189	3.104146	C	6.912952	-2.977435	1.589412	C	-1.588673	-8.590213	-2.196150
H	-1.151719	11.518627	2.599283	C	2.578748	-4.044737	1.339639	H	-2.465528	-6.865421	-1.311769
H	-1.214008	10.700982	4.195432	C	-10.053964	5.150831	-3.445363	C	0.830435	-8.710489	-2.190528
H	-0.188413	12.145369	3.966460	C	-3.356132	-3.489904	-1.034605	H	1.849268	-7.066391	-1.294657
C	-5.714659	3.641715	-1.261499	H	-4.030220	-3.439663	-3.093752	C	-0.407373	-9.283831	-2.526103
C	-4.711953	1.575131	-0.303017	C	-7.144727	-2.352235	1.597814	H	-2.541025	-9.050656	-2.437615
C	-4.701259	1.169146	1.051272	C	-6.052720	-4.508836	1.545446	H	1.758635	-9.225966	-2.407102
H	-4.983834	1.793807	3.108175	C	-2.921412	-3.806328	1.341651	O	-0.574750	-10.472518	-3.139211
C	7.144726	2.352238	-1.597816	H	9.441129	6.764383	-2.508619	C	0.574531	-11.244399	-3.490009
C	6.052713	4.508836	-1.545449	H	8.713214	6.450090	-4.117485	H	1.151754	-11.518621	-2.599279
C	4.783690	0.206159	-1.323846	H	10.474198	6.271421	-3.879356	H	1.214034	-10.700978	-4.195430
C	4.750662	-0.622626	1.319925	C	1.832127	-4.437311	-1.300758	H	0.188449	-12.145371	-3.966454

N-Belt **2**$^{3+}$ (UB3LYP/6-31G(d)_Q) E = -4248.25556306

N	-0.746266	-5.474847	0.613510	C	5.130161	-5.518053	-1.540318	C	-6.111401	6.278346	2.139385
C	-0.916576	-6.713489	1.240689	C	6.624266	-3.604533	-1.537616	H	-4.185342	5.983803	1.290095
C	-1.844606	-4.612141	0.298739	C	4.680233	-1.061341	-1.318892	C	-7.612338	4.368707	2.139997
C	-2.212077	-7.201778	1.550630	C	4.789425	-0.227163	1.323602	H	-6.846259	2.575221	1.284714
C	0.191967	-7.539956	1.538024	C	-8.490249	-2.156830	-2.152846	C	-0.908263	4.753639	1.053976
C	0.543063	-4.937833	0.299977	H	-7.273357	-0.633849	-1.305841	H	-2.515111	4.138242	2.349455
C	-2.594042	-4.033675	1.321959	C	-7.588634	-4.412170	-2.142884	H	10.098967	-5.754820	-2.493391
C	-2.147608	-4.339469	-1.055610	H	-5.655027	-4.643749	-1.281136	H	9.492685	-5.256231	-4.110012
C	-2.377903	-8.432101	2.149476	C	-4.570696	1.589188	-1.059221	H	10.058570	-6.929274	-3.839156
H	-3.088637	-6.615769	1.304753	H	-4.840103	-0.110265	-2.354621	C	2.594042	4.033675	-1.321959
C	0.026214	-8.778079	2.140179	C	-4.548909	1.997917	0.294679	C	8.490249	2.156830	2.152846
H	1.193386	-7.217750	1.280957	H	-4.641158	1.400483	2.349857	H	7.273357	0.633849	1.305841
C	1.419210	-4.581712	1.324170	C	6.111401	-6.278346	-2.139385	C	7.588634	4.412170	2.142884
C	0.908263	-4.753639	-1.053976	H	4.185342	-5.983803	-1.290095	H	5.655027	4.643749	1.281136
C	-3.664326	-3.163774	1.056152	C	7.612338	-4.368707	-2.139997	C	2.147608	4.339469	1.055610
H	-2.328191	-4.245913	2.353321	H	6.846259	-2.575221	-1.284714	H	3.532288	3.319897	2.352749
C	-3.259174	-3.522987	-1.321441	C	4.832123	0.309773	-1.054516	C	-0.543063	4.937833	-0.299977
C	-1.339969	-4.833092	-2.169065	H	4.641158	-1.400483	-2.349857	H	-1.105660	4.716650	-2.355148
C	-1.263621	-9.241641	2.464031	C	4.916461	0.709955	0.299419	C	-7.368541	5.718601	2.458999
H	-3.368173	-8.808928	2.382619	H	4.840103	0.110265	2.354621	H	-5.942470	7.325199	2.368611
H	0.900503	-9.384022	2.346106	C	-8.634177	-3.526446	-2.468098	H	-8.573477	3.914779	2.350069
C	2.682378	-4.027331	1.059642	H	-9.311212	-1.487311	-2.386834	C	0.000512	5.014292	2.168436
H	1.105660	-4.716650	2.355148	H	-7.676349	-5.472229	-2.349123	C	1.844606	4.612141	-0.298739
C	2.197060	-4.261781	-1.318430	C	-4.340142	2.506039	-2.173711	H	2.328191	4.245913	-2.353321
C	-0.000512	-5.014292	-2.168436	N	-4.368613	3.382968	0.607939	C	8.634177	3.526446	2.468098
C	-4.005975	-2.941069	-0.298146	C	7.368541	-5.718601	-2.458999	H	9.311212	1.487311	2.386834
C	-4.344581	-2.505699	2.169723	H	5.942470	-7.325199	-2.368611	H	7.676349	5.472229	2.349123
H	-3.532288	-3.319897	-2.352749	H	8.573477	-3.914779	-2.350069	C	1.339969	4.833092	2.169065
H	-1.863456	-4.957087	-3.114431	C	4.856689	1.254817	-2.168960	N	0.746266	5.474847	-0.613510
O	-1.542355	-10.415505	3.046527	N	5.115171	2.092709	0.612866	O	-8.244606	6.548430	3.041139
C	3.071610	-3.901752	-0.294305	O	-9.789342	-3.871373	-3.052509	H	-0.472205	5.273112	3.113254
C	3.512625	-3.574771	2.173863	C	-3.512625	3.574771	-2.173863	O	9.789342	3.871373	3.052509
H	2.515111	-4.138242	-2.349455	H	-4.799794	2.226044	-3.118940	H	1.863456	4.957087	3.114431
H	0.472205	-5.273112	-3.113254	C	-5.355262	4.150881	1.235291	C	0.916576	6.713489	-1.240689
N	-5.115171	-2.092709	-0.612866	C	-3.071610	3.901752	0.294305	C	-9.553239	6.076860	3.386914
C	-4.856689	-1.254817	2.168960	O	8.244606	-6.548430	-3.041139	C	10.035157	5.241431	3.394603
H	-4.332932	-3.043590	3.115065	C	4.344581	2.505699	-2.169723	C	2.212077	7.201778	-1.550630
C	-0.478618	-11.313584	3.387722	H	5.225875	0.862300	-3.113820	C	-0.191967	7.539956	-1.538024

N	4.368613	-3.382968	-0.607939
C	4.340142	-2.506039	2.173711
H	3.356789	-4.089579	3.119281
C	-6.272520	-2.564070	-1.241316
C	-4.916461	-0.709955	-0.299419
C	-4.832123	-0.309773	1.054516
H	-5.225875	-0.862300	3.113820
H	0.069720	-11.624753	2.491952
H	0.204591	-10.851498	4.108604
H	-0.962744	-12.177580	3.841179
C	5.355262	-4.150881	-1.235291
C	4.548909	-1.997917	-0.294679
C	4.570696	-1.589188	1.059221
H	4.799794	-2.226044	3.118940
C	-7.342519	-1.685852	-1.552283
C	-6.434143	-3.937218	-1.539065
C	-4.789425	0.227163	-1.323602
C	-4.680233	1.061341	1.318892
C	6.272520	2.564070	1.241316
C	4.005975	2.941069	0.298146
C	-10.035157	-5.241431	-3.394603
C	-2.682378	4.027331	-1.059642
H	-3.356789	4.089579	-3.119281
C	-5.130161	5.518053	1.540318
C	-6.624266	3.604533	1.537616
C	-2.197060	4.261781	1.318430
C	9.553239	-6.076860	-3.386914
C	3.664326	3.163774	-1.056152
H	4.332932	3.043590	-3.115065
C	7.342519	1.685852	1.552283
C	6.434143	3.937218	1.539065
C	3.259174	3.522987	1.321441
H	-10.032337	-5.872178	-2.499029
H	-9.292217	-5.602126	-4.114260
H	-11.024648	-5.253622	-3.849848
C	-1.419210	4.581712	-1.324170
H	-10.098967	5.754820	2.493391
H	-9.492685	5.256231	4.110012
H	-10.058570	6.929274	3.839156
H	10.032337	5.872178	2.499029
H	9.292217	5.602126	4.114260
H	11.024648	5.253622	3.849848
C	2.377903	8.432101	-2.149476
H	3.088637	6.615769	-1.304753
C	-0.026214	8.778079	-2.140179
H	-1.193386	7.217750	-1.280957
C	1.263621	9.241641	-2.464031
H	3.368173	8.808928	-2.382619
H	-0.900503	9.384022	-2.346106
O	1.542355	10.415505	-3.046527
C	0.478618	11.313584	-3.387722
H	-0.069720	11.624753	-2.491952
H	-0.204591	10.851498	-4.108604
H	0.962744	12.177580	-3.841179

7. References

1 M. Kuprat, M. Lehmann, A. Schulz and A. Villinger, *Organometallics* 2010, **29**, 1421.

2 G. Povie, Y. Segawa, T. Nishihara, Y. Miyauchi and K. Itami, *Science* 2017, **356**, 172–175.

3 G. M. Sheldrick, *Acta Crystallogr.* 2015, **A71**, 3-8.

4 G. M. Sheldrick, *Acta Crystallogr.* 2015, **C71**, 3-8.

5 O. V. Dolomanov, L. J. Bourhis, R. J. Gildea, J. A. K. Howard and H. Puschmann, *J. Appl. Crystallogr.* 2009, **42**, 339–341.

6 M. J. Frisch, G. W. Trucks, H. B. Schlegel, G. E. Scuseria, M. A. Robb, J. R. Cheeseman, G. Scalmani, V. Barone, G. A. Petersson, H. Nakatsuji, X. Li, M. Caricato, A. V. Marenich, J. Bloino, B. G. Janesko, R. Gomperts, B. Mennucci, H. P. Hratchian, J. V. Ortiz, A. F. Izmaylov, J. L. Sonnenberg, D. Williams-Young, F. Ding, F. Lipparini, F. Egidi, J. Goings, B. Peng, A. Petrone, T. Henderson, D. Ranasinghe, V. G. Zakrzewski, J. Gao, N. Rega, G. Zheng, W. Liang, M. Hada, M. Ehara, K. Toyota, R. Fukuda, J. Hasegawa, M. Ishida, T. Nakajima, Y. Honda, O. Kitao, H. Nakai, T. Vreven, K. Throssell, J. A. Montgomery, Jr., J. E. Peralta, F. Ogliaro, M. J. Bearpark, J. J. Heyd, E. N. Brothers, K. N. Kudin, V. N. Staroverov, T. A. Keith, R. Kobayashi, J. Normand, K. Raghavachari, A. P. Rendell, J. C. Burant, S. S. Iyengar, J. Tomasi, M. Cossi, J. M. Millam, M. Klene, C. Adamo, R. Cammi, J. W. Ochterski, R. L. Martin, K. Morokuma, O. Farkas, J. B. Foresman and D. J. Fox, Gaussian 16, Revision C.01, Gaussian, Inc., Wallingford CT, 2019.

7 (a) A. D. Becke, *J. Chem. Phys.* 1993, **98**, 5648. (b) C. Lee, W. Yang, R. G. Parr, *Phys. Rev. B* 1988, **37**, 785–789.
